# Targeting aberrant sialylation and fucosylation in prostate cancer cells using potent metabolic inhibitors

**DOI:** 10.1093/glycob/cwad085

**Published:** 2023-10-17

**Authors:** Margarita Orozco-Moreno, Eline A Visser, Kirsty Hodgson, Agnes L Hipgrave Ederveen, Kayla Bastian, Emily Archer Goode, Özden Öztürk, Johan F A Pijnenborg, Nienke Eerden, Sam J Moons, Emiel Rossing, Ning Wang, Noortje de Haan, Christian Büll, Thomas J Boltje, Jennifer Munkley

**Affiliations:** Newcastle University Centre for Cancer, Newcastle University Institute of Biosciences, Central Parkway, Newcastle-upon-Tyne, Tyne and Wear NE1 3BZ, United Kingdom; Synthetic Organic Chemistry, Institute for Molecules and Materials, Radboud University Nijmegen, Toernooiveld 1, 6525 ED Nijmegen, The Netherlands; Newcastle University Centre for Cancer, Newcastle University Institute of Biosciences, Central Parkway, Newcastle-upon-Tyne, Tyne and Wear NE1 3BZ, United Kingdom; Center for Proteomics and Metabolomics, Leiden University Medical Center, Albinusdreef 2, 2333 ZA Leiden, The Netherlands; Newcastle University Centre for Cancer, Newcastle University Institute of Biosciences, Central Parkway, Newcastle-upon-Tyne, Tyne and Wear NE1 3BZ, United Kingdom; Newcastle University Centre for Cancer, Newcastle University Institute of Biosciences, Central Parkway, Newcastle-upon-Tyne, Tyne and Wear NE1 3BZ, United Kingdom; Synthetic Organic Chemistry, Institute for Molecules and Materials, Radboud University Nijmegen, Toernooiveld 1, 6525 ED Nijmegen, The Netherlands; GlycoTherapeutics B.V., Toernooiveld 1, 6525 ED Nijmegen, The Netherlands; Synthetic Organic Chemistry, Institute for Molecules and Materials, Radboud University Nijmegen, Toernooiveld 1, 6525 ED Nijmegen, The Netherlands; GlycoTherapeutics B.V., Toernooiveld 1, 6525 ED Nijmegen, The Netherlands; Synvenio B.V., Toernooiveld 1, 6525 ED Nijmegen, The Netherlands; Synthetic Organic Chemistry, Institute for Molecules and Materials, Radboud University Nijmegen, Toernooiveld 1, 6525 ED Nijmegen, The Netherlands; The Mellanby Centre for Musculoskeletal Research, Department of Oncology and Metabolism, The University of Sheffield, Medical School, Beech Hill Rd, Sheffield, Yorkshire S10 2RX, United Kingdom; Center for Proteomics and Metabolomics, Leiden University Medical Center, Albinusdreef 2, 2333 ZA Leiden, The Netherlands; Biomolecular Chemistry, Institute for Molecules and Materials, Radboud University Nijmegen, Heyendaalseweg 135, 6525 AJ Nijmegen, The Netherlands; Synthetic Organic Chemistry, Institute for Molecules and Materials, Radboud University Nijmegen, Toernooiveld 1, 6525 ED Nijmegen, The Netherlands; Newcastle University Centre for Cancer, Newcastle University Institute of Biosciences, Central Parkway, Newcastle-upon-Tyne, Tyne and Wear NE1 3BZ, United Kingdom

**Keywords:** fucosylation, glycans, metabolic inhibitors, prostate cancer, sialylation

## Abstract

Aberrant glycosylation is a hallmark of cancer and is not just a consequence, but also a driver of a malignant phenotype. In prostate cancer, changes in fucosylated and sialylated glycans are common and this has important implications for tumor progression, metastasis, and immune evasion. Glycans hold huge translational potential and new therapies targeting tumor-associated glycans are currently being tested in clinical trials for several tumor types. Inhibitors targeting fucosylation and sialylation have been developed and show promise for cancer treatment, but translational development is hampered by safety issues related to systemic adverse effects. Recently, potent metabolic inhibitors of sialylation and fucosylation were designed that reach higher effective concentrations within the cell, thereby rendering them useful tools to study sialylation and fucosylation as potential candidates for therapeutic testing. Here, we investigated the effects of global metabolic inhibitors of fucosylation and sialylation in the context of prostate cancer progression. We find that these inhibitors effectively shut down the synthesis of sialylated and fucosylated glycans to remodel the prostate cancer glycome with only minor apparent side effects on other glycan types. Our results demonstrate that treatment with inhibitors targeting fucosylation or sialylation decreases prostate cancer cell growth and downregulates the expression of genes and proteins important in the trajectory of disease progression. We anticipate our findings will lead to the broader use of metabolic inhibitors to explore the role of fucosylated and sialylated glycans in prostate tumor pathology and may pave the way for the development of new therapies for prostate cancer.

## Introduction

Prostate cancer is the most frequently diagnosed cancer among men and is the cause of over 350,000 deaths worldwide every year (Siegel et al. 2022). Organ confined prostate cancer is managed through surgery or localized radiation therapy, but for patients who recur following treatment or in advanced or metastatic disease the mainstay therapy is androgen deprivation therapy (ADT) (Desai et al. 2021). Unfortunately, ADT leads to the emergence of resistance mechanisms and longer term the disease progresses to castrate resistant prostate cancer (CRPC) which is ultimately fatal (Karen et al. 2016). When detected at an early stage, the 5-year survival rate for prostate cancer is close to 100%, but if patients are diagnosed with late stage or metastatic disease this decreases to 33% (Siegel et al. 2023). The progression from a hormone dependent state to an ADT resistant phenotype involves changes in androgen receptor (AR) signaling and associated pathways, and the most common sites for metastasis are lymph nodes, bone, lung, and liver (Wilt and Ahmed 2013). New therapies for prostate cancer are urgently needed and could significantly improve quality of life and survival times for patients.

It is well documented that the development and progression of prostate cancer is associated with fundamental changes in the glycosylation patterns of both cell surface and secreted glycoproteins (Munkley, Mills, et al. 2016; Munkley, Vodak, et al. 2016; Munkley 2017; Scott and Munkley 2019). Two key changes in glycosylation frequently detected in prostate cancer are aberrant fucosylation and sialylation, with qualitative changes to fucosylated and sialylated glycans believed to play a fundamental role in disease pathology (Scott and Munkley 2019; Butler and Huang 2021; Kaluza et al. 2021). Fucosylation is the attachment of a fucose residue to a glycan and consists of core fucosylation and terminal fucosylation (Shan et al. 2019). Sialylation is the addition of sialic acid residues to glycans as the terminal monosaccharide (Munkley and Scott 2019). Both increased fucosylation and increased levels of sialic acid have been detected in the serum of patients with prostate cancer and are associated with aggressive disease (Ohyama et al. 2004; Kyselova et al. 2007; Saldova et al. 2011; Fujita et al. 2014; Scott and Munkley 2019; Zhang et al. 2019). Studies have also linked overexpression of fucosyltransferase and sialyltransferase enzymes that underpin the biosynthesis of these malignant glycans to prostate cancer progression. These include, upregulation of the fucosyltransferase FUT6 which promotes prostate cancer metastasis to bone (Li et al. 2013), the overexpression of the core fucosyltransferase FUT8 which can support cell proliferation in castrated conditions (Hoti et al. 2018), increased expression of the sialyltransferase ST6GAL1 which is linked to aggressive prostate cancer cell behavior (Wei et al. 2016; Scott, Archer Goode, et al. 2023), and upregulation of ST6GALNAC1 which regulates prostate cancer cell adhesion (Munkley et al. 2015). Numerous studies have also reported the upregulation of specific sialylated and fucosylated glycans in prostate cancer, including the Sialyl Lewis X (sLe^X^) antigen and the cancer associated sialyl-Tn glycan (known as sTn) which are linked to poor prognosis in patients through various mechanisms (Munkley 2016; Butler and Huang 2021).

**Fig. 1:**
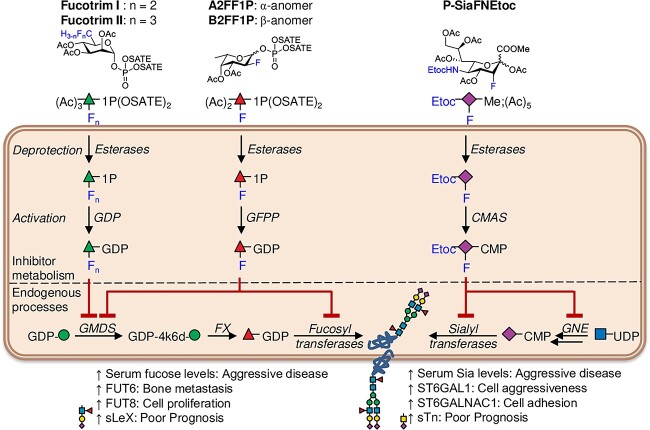
Mechanisms of action of the fucosylation and sialylation inhibitors Fucotrim I, Fucotrim II, A2FF1P, B2FF1P and P-SiaFNEtoc. Fucotrim I and II are Rhamnose-1-phosphate derivatives and are designed to enter the de novo fucose biosynthesis pathway to target the crucial GMDS enzyme. As Fucotrim I and II are designed to mimic mannose-1-phosphate it has been observed that these compounds also lower the GDP-mannose levels. A2FF1P and B2FF1P are derived from 2-fluorofucose (2FF) and are metabolic inhibitors of fucosyltransferases. They are metabolically processed via the fucose salvage pathway leading to GDP-2-fluorofucose, which is a competitive inhibitor of fucosyltransferases and additionally halts the de novo biosynthesis of GDP-fucose via feedback-inhibition. The C-5-modified 3-fluoro sialic acid sialyltransferase inhibitor P-SiaFNEtoc has the natural N-acetamide group replaced with a carbamate functionality and reaches higher effective concentrations within the cell to inhibit sialic acid incorporation. Monosaccharide symbols follow the SNFG (Symbol Nomenclature for Glycans) system (Varki et al. 2015).

Due to their fundamental roles in cancer, glycans and their cognate receptors are emerging as novel targets for drug discovery and development (Mereiter et al. 2019; Costa et al. 2020). A repertoire of fucosyltransferase inhibitors has been developed employing a variety of strategies (Bastian et al. 2021). The orally bioavailable cell permeable fucosylation inhibitor 2-fluorofucose (SGN-2FF) has shown promising anti-cancer effects both in vitro and in vivo, including for prostate cancer, and shows direct and indirect effects on immune cells, tumor cells and the tumor microenvironment (Li et al. 2013; Okeley et al. 2013; Zhou et al. 2017; Carrascal et al. 2018; Okeley et al. 2018; Disis et al. 2020; Bastian et al. 2021). SGN-2FF has been tested in a Phase I clinical trial for patients with advanced solid tumors, where it demonstrated potential to suppress tumor growth, but the study had to be terminated after three years due to safety concerns (NCT 02952989) (Do et al. 2021). The efficacy of SGN-2FF and its promise as a cancer therapeutic has inspired the development of a number of additional fucosylation inhibitors (Rossing et al. 2022). These include, the SGN-2FF derivatives A2FF1P and B2FF1P (Pijnenborg, Visser, et al. 2021) and the metabolic inhibitors Fucotrim I and Fucotrim II (Pijnenborg, Rossing, et al. 2021) (Fig. 1).

The targeting of aberrant sialylation is also being explored as a strategy to develop new cancer therapies. The sialyltransferase inhibitor P-3F_AX_-Neu5Ac has been shown to inhibit all sialyltransferase enzymes and can reduce global sialylation by up to 80% (Rillahan et al. 2012; Büll et al. 2013), however, when P-3F_AX_-Neu5Ac was tested in a mouse model, systemic delivery of this sialylation inhibitor produced liver and kidney dysfunction (Macauley et al. 2014). Büll et al. have since utilized targeted delivery of P-3F_AX_-Neu5Ac using nanoparticles to inhibit metastasis (Büll et al. 2015) and localized administration through intra-tumoral injections to suppress the growth of multiple tumor types by promoting T-cell mediated immunity (Büll et al. 2018). Although a narrow therapeutic window was identified at which antitumor effects were observed without adverse side effects, at higher doses renal toxicity was induced, highlighting the need for better tolerated sialyltransferase inhibitors for systemic use (Munkley 2022). Recently, novel C-5-modified 3-fluoro sialic acid sialyltransferase inhibitors (where the natural N-acetamide group is replaced with a carbamate functionality) have been developed that are more efficiently metabolized and reach higher effective concentrations within the cell (Heise et al. 2019; Moons et al. 2022) (Fig. 1). This expanding toolbox of inhibitors with increased potency of both fucosylation and sialylation inhibition enables the investigation of their future therapeutic potential in the context of prostate cancer progression.

Here, we explore the use of a panel of potent metabolic inhibitors of fucosylation and the C-5 carbamate sialyltransferase inhibitor P-SiaFNEtoc as a new class of drugs for prostate cancer. Our findings show these inhibitors shut down the synthesis of fucosylated and sialylated glycans and remodel the prostate cancer cell surface glycome within days. Using in vitro assays, we found that treatment with inhibitors Fucotrim I and Fucotrim II inhibits prostate cancer cell proliferation and induces apoptosis, and treatment with the sialylation inhibitor P-SiaFNEtoc reduces the growth of prostate cancer cells in colonies. Furthermore, using RNA-sequencing, we reveal that these inhibitors regulate oncogenic gene expression patterns important in prostate tumor progression, and using lectin and mass spectrometry profiling methods we show they are very specific for their respective family of target glycosyltransferases and have minor side effects on other glycosylation steps. Our data identifies potent metabolic inhibitors of fucosylation and sialylation as promising new treatments for prostate cancer (once modifications render them more targeted toward cancer cells) and points to the need for further studies in this area.

## Materials and methods

### Inhibitors

P-SiaFNEtoc (compound 10 in (Heise et al. 2019)), 2FF, A2FF1P and B2FF1P (Pijnenborg, Visser, et al. 2021), and Fucotrim I and II (Pijnenborg, Rossing, et al. 2021) were synthesized as described previously. SGN-2FF was purchased from Cambridge Bioscience (HY-107366).

### Cell culture

Culture of cells was as described previously (Munkley et al. 2019). PC3 and CWR22RV1 cells were obtained from ATCC (CRL-1435 and CRL-2505). PNT2 and BPH-1 cells were purchased from Sigma (95012613 and SCC256). All cells were cultured at 37 °C, 5% CO2 in a humidified incubator, and passaged with trypsin every 3–4 days. Cells were passaged until a maximum passage number of 30–35. Cell lines were authenticated using DNA STR analysis and tested every 3 months for mycoplasma contamination.

### Flow cytometry using lectins and Lectenz

Cells were cultured in medium containing different concentrations of unnatural sugar derivatives in 48-wells plates (50,000 cells per well for PC3 cells, and 250,000 cells per well for CWR22RV1 cells) seeded the day before the experiment. DMSO, at same dilution as the unnatural sugar derivative stock solutions, was used as a positive control for lectin staining. Biotinylated AAL (B-1395), SNA (B-1305), MAL-II (B-1265), WGA (B-1025), LCA (B-1045), PNA (B-1075) and PHA-L (B-1115) lectins were purchased from Vector laboratories, and biotinylated AOL lectin (A2659) was purchased from TCI Europe. Cells were incubated with the inhibitors for three days (at the concentration indicated), after which cells were harvested with trypsin and centrifugation (2,000 rpm at 4 °C). The cells were then washed with 100 μL PBS and then resuspended in 50 μL of 5 μg/mL biotinylated lectin in 1X Carbo-free blocking buffer (Vector Labs, SP-5040) containing 1 mM CaCl_2_ and 1 mM MgCl_2_ (1xCF) and incubated at 4 °C for 60 min. Cells were washed three times with 100 μL PBA (PBS containing 1% v/v FBS and 0.02% w/w sodium azide) and subsequently incubated with 40 μL 0.8 μg/mL Streptavidin-phycoerythrin conjugate (Invitrogen, 12-4317-87) in PBA for 10 min at 4 °C. Cells were then washed again three times with 100 μL PBA, resuspended in 50–100 μL PBA and fluorescence was measured with a flow cytometer (Beckman & Dickinson FACSCalibur (Beckman & Coulter Cytoflex)). Each replicate for each condition had >10,000 gated events. Data was processed using FlowJo V10 (FlowJo LLC). The percentage of lectin binding was obtained by normalizing the MFI values to the MFI values of the respective DMSO control. For the Lectenz, the protocol mostly followed the one above. The only difference is that a pre-incubation was made of 2 μg/mL Pan-specific Lectenz (Lectenz bio, SK0501B) with 0.8 μg/mL Strep-PE in 1xCF for at least 10 min before use. After 1 wash with PBA, cells were incubated with the Lectenz/Strep-PE for 1 h at 4 °C. After three washes with PBA, the cells were resuspended in PBA and measured with the flow cytometer. The protocol below was repeated until *n* > 3 for each compound using cells at different passage numbers on different days.

### Lectin immunofluorescence

Cells were cultured in Lab-Tek™II Chamber Slides (Thermo Scientific, 154453) for 72 h in complete media containing DMSO (vehicle control) or 2 μM P-SiaFNEtoc. Cells were washed with PBS before permeabilization and fixation with ice-cold absolute methanol for 10 min at −20 °C. Next, slides were washed with PBS and blocked with Carbo-Free™ Blocking solution (Vector Laboratories, SP-5040) for 1 h at room temperature. Slides were incubated overnight at 4 °C with FITC-conjugated SNA lectin (Vector labs, FL-1301-2) or FITC-conjugated MAL-I lectin (Vector labs, FL-1311-2) at 1:200. Finally, slides were washed with PBS and stained with Hoechst (Thermo Scientific, 62249) for 15 min at room temperature. Cells were mounted using ProLong™ gold antifade mountant (Thermo Fisher, P36930). Images were acquired and processed with the ZEISS Axio Imager 4.

### Immunocytochemistry

Cells were cultured in Lab-Tek™II Chamber Slides (Thermo Scientific, 154453,) for 72 h in complete media containing DMSO (vehicle control) or 2 μM P-SiaFNEtoc. Cells were washed with PBS before permeabilization and fixation with ice-cold absolute methanol for 10 min at −20 °C. Next, slides were washed with PBS and blocked with 10% goat serum (Abcam, ab7481) for 1 h at room temperature. Slides were incubated overnight at 4 °C with p27/Kip1 (Cell Signaling, 3686) at 1:1000. Finally, slides were washed with PBS and stained with Hoechst (Thermo Scientific, 62249) for 15 min at room temperature. Images were acquired and processed with the ZEISS Axio Imager 5. The fluorescent images were analyzed using Fiji software (Schindelin et al. 2012) by measuring the area, integrated density and mean gray value of one cell at a time (*n* = 150). The fluorescence intensity was calculated in Excel using the formula for corrected total cell fluorescence (CTCF) = integrated density–(area of selected cell × mean fluorescence of background readings).

### In vitro cell behavior assays

WST-1 cell proliferation assays were carried out as previously described (Scott et al. 2022; Scott, Hodgson, et al. 2023). For the colony formation assays cells were drugged at the indicated concentrations for 72 h with their respective DMSO controls, then 100 cells/well were seeded in 6-well dishes with the treatment and maintained for 14 days until colonies of more than 50 cells/colony had formed. The culture medium and treatment were changed on day 7. Colonies were fixed with ice-cold absolute methanol for 10 min then stained with 0.5% w/v crystal violet. For CellTiter-Glo® assays, cells were seeded in Nunc™ MicroWell™ 96-well plates (Thermo Scientific, 10072151) at 10,000 cells per well in 100 μL complete media. After 24 h, cells were drugged at the range of concentrations indicated. Cell viability was assessed at 72 h with the CellTiter-Glo® Luminescent Cell Viability Assay (Promega, G7571). Luminescence was recorded with the Varioskan™ LUX microplate reader. Dose response curves, unpaired t-tests and bar plots were generated using GraphPad PRISM version 9.5.0. Data are presented as the mean of three biological repeats ± standard error of the mean (SEM). At least 3 technical and 3 biological repeats were performed for each experiment, with representative data shown in the figures.

### RNA-sequencing

RNA was extracted from CWR22RV1 cells treated with DMSO, 2 μM P-SiaFNEtoc, 100 μM SGN-2FF, 30 μM Fucotrim I or 60 μM Fucotrim II for 72 h with 3 biological repeats for each condition. Samples were prepared with the Illumina TruSeq Stranded mRNA Library Prep Kit and sequenced using an Illumina NovaSeq 6000, giving >20 million 100 bp single reads per sample. All data analyses were performed in Galaxy version 22.01. Quality control was performed with FastQC (http://www.bioinformatics.babraham.ac.uk/projects/fastqc/) and reads were trimmed with Cutadapt (Martin 2011). Reads were mapped to hg38 using HISAT2 (Kim et al. 2015) and quantified with featureCounts (Liao et al. 2014). Differential gene expression analysis was performed using limma-voom (Law et al. 2014) and volcano plots were generated with ggplot2 (Wickham 2016). Gene Set Enrichment Analysis (GSEA) was performed with the package EGSEA (Alhamdoosh et al. 2017) and a heatmap was generated with ggplot2. RNA-Seq data can be accessed on GEO repository (GSE232621).

### Oncology array

CWR22RV1 cells were cultured in T75 flasks in complete media containing DMSO (vehicle control) or 2 μM P-SiaFNEtoc for 72 h. Cell lysates were incubated with 600 μL of Lysis Buffer 17 (895943, R&D Systems) at 4 °C for 30 min and concentration was determined with the Pierce™ 660 nm Protein Assay Kit (22662, Thermo Scientific). For conditioned media samples, cells were cultured in serum free media containing DMSO (vehicle control) or 2 μM P-SiaFNEtoc for an additional 48 h then conditioned media was collected in Amicron® Ultra-15 Centrifugal Filter Units (UFC901024, Millipore). The Proteome Profiler Human XL Oncology Array (ARY026, R&D Systems) was performed as per the manufacturer’s instructions, using 200 μg cell lysate or 330 μL conditioned media per sample.

### Apoptosis assays

Cells were seeded in T25 culture flasks one day prior to treatment with either DMSO, Fucotrim I or Fucotrim II at the range of concentrations indicated for 72 h. For the fucose rescue apoptosis assays, 100 μM L-fucose (Thermo Scientific, A16789.03) was added simultaneously with the treatment. Apoptosis assays were performed using the eBioscience™ Annexin V Apoptosis Detection Kit (Thermo Scientific, BMS500FI-300) according to the manufacturer’s instructions. The stained samples were analyzed on a BD LSRFortessa™ Cell Analyzer (BD Biosciences). Apoptosis rates were determined by the sum of early and late apoptosis rates (Annexin V staining only = early apoptosis, Annexin V and Propidium Iodide co-staining = late apoptosis), while necrosis was defined as Propidium Iodide staining only. Data was analyzed using the FCS Express™ Flow Cytometry Analysis Software. Data are presented as the mean of three biological repeats ± standard error of the mean (SEM).

### ELISA assays

Pre-validated ELISA assays for human VEGF (Abcam, ab100662) and human DKK-1 (Cambridge Bioscience, ELH-DKK1-1) were carried out using conditioned media samples as per the manufacturer’s instructions.

### Protein blotting and glycan release

PC3 cells were seeded at 320,000 cells/well in a 6-well plate one day prior to addition of fresh medium with either P-SiaFNEtoc (2 μM), A2FF1P (100 μM), B2FF1P (100 μM), Fucotrim I (30 μM), Fucotrim II (60 μM), DMSO (similar volume), Combination of P-SiaFNEtoc and B2FF1P, Combination of P-SiaFNEtoc and Fucotrim I, Combination of Fucotrim I and L-fucose (100 μM); and Combination of DMSO and L-fucose. After an incubation of 72 h, cells were washed carefully three times with 2.5 mL PBS. Subsequently, cells were resuspended in 1 mL PBS, transferred to 1.5 mL Eppendorf tubes and harvested by centrifugation (300*g* at 4 °C). Cell pellets were washed once with 500 μL PBS, after which they were snap-frozen in liquid nitrogen and stored in a −80 °C freezer until further use.

Cell lysate proteins (approximately 500,000 cells/well) were blotted on the polyvinylidenefluoride (PVDF) membranes (MultiScreenHTS IP Filter Plate, 0.45 μm, Millipore) as described previously (Zhang et al. 2020). Briefly, *N*-glycans were enzymatically released using 2 U PNGase F, eluted with water after overnight incubation at 37 °C and dried in a vacuum concentrator. Subsequentially, a nonreductive *O*-glycan release as described previously was conducted on the de-N-glycosylated proteins on the PVDF membrane (de Haan et al. 2022). The release agent was diluted to 25 μL containing 20% hydroxylamine and 20% 1,8-diazabicyclo(5.4.0)undec-7-ene (DBU) in water and incubated for 75 min at 37 °C in a moisture box.

### *N*-glycan linkage-specific sialic acid esterification, reducing end labeling and HILIC SPE

The ethyl esterification derivatization was performed by resuspending the dried *N*-glycans in 2 μL water and adding 20 μL of ethyl esterification reagent (0.25 M 1-Ethyl-3-(3-(dimethylamino)propyl)carbodiimide (EDC) hydrochloride with 0.25 M 1-hydroxybenzotriazole in ethanol) and incubating the mixture for 30 min at 37 °C (Reiding et al. 2014), after 30 min 4 μL of 28% ammonia solution was added and incubated for an additional 30 min. In-house assembled microtips used for cotton hydrophilic interaction chromatography (HILIC) solid phase extraction (SPE) microtip purification were prepared as described (Selman et al. 2011). The glycans were purified by cotton HILIC SPE and eluted in water. The N-glycans were diluted to 25 μL 10% acetic acid. 25 μL of 2-AB reagent (1 M 2-AB, 232 mM 2-methylpyridine borane complex in 90:10 metanol:acetic acid) was added and the 2-AB labeling reaction was incubated for 2.5 h at 50 °C, 1 mL of ACN was added, and the glycans were purified by HILIC SPE and eluted in 50 μL of water.

### *O*-glycan recovery, reducing end labeling and SPE

The *O*-glycans were recovered from the PVDF membrane by centrifugation and 1 mL of acetonitrile (ACN) containing 2 mg of magnetic hydrazide beads (MagSi-S Hydrazide beads 1 μm, magtivio B.V., Nuth, The Netherlands) was added. After two washes with ACN, the *O*-glycans were eluted from the hydrazide beads in 50 μL of 2-AB reagent (500 mM 2-AB, 116 mM 2-methylpyridine borane complex in 45:45:10 methanol:water:acetic acid). The 2-AB labeling reaction was incubated as described above and purified by cotton HILIC SPE and eluted in water. The labeled *O*-glycans were further purified using porous graphitic carbon (PGC) SPE as described (Zhang et al. 2020). The samples were dried and reconstituted in 20 μL of water for MS analysis.

### Mass spectrometry

One microliter per sample was injected per analysis. The glycans were separated by nanoflow liquid chromatography (nanoLC), mobile phase A was 0.1% formic acid in MQ. A gradient from 2% to 38% mobile phase B in 20 min (0.1% formic acid/80% acetonitrile) was used for elution of glycans. A single analytical column setup packed with Reprosil-Pure-AQ C18 phase (Dr. Maisch, 1.9 μm in particle size, 30 cm in column length) in an EASY-nLC 1,200 UHPLC (Thermo Fisher Scientific) using a PicoFrit Emitter (New Objectives, 75 μm in inner diameter). The emitter was interfaced to an Orbitrap Fusion Lumos MS (Thermo Fisher Scientific) via a nanoSpray Flex ion source. A precursor MS scan (*m/z* 275–1,700, positive polarity) was acquired in the Orbitrap at a nominal resolution of 120,000, followed by Orbitrap higher-energy C-trap dissociation (HCD)-MS/MS at a nominal resolution of 30,000 of the 10 most abundant precursors in the MS spectrum (charge states 1 to 4). A minimum MS signal threshold of 50,000 was used to trigger data-dependent fragmentation events. HCD was performed with an energy of 27% ± 5%, applying a 10 s dynamic exclusion window. Data analysis was performed as described (de Haan et al. 2022). Briefly, MS1 feature detection in the raw files was performed using the Minora Feature Detector node in Thermo Proteome Discoverer 2.5.0.400. The [M + H] values of the resulting features were imported into GlycoWorkbench 2.1 (build 146) 7 and matched to glycan compositions. The complete list of identified compositions was imported into Skyline 22.2.0.351 (ProteoWizard), using the Molecule Interface. Extracted ion chromatograms were generated for the first three isotopologues of each glycan. Chromatographic peaks were manually selected based on accurate mass (>−1 ppm, <1 ppm) and isotopic dot product (idotp; > 0.80) in at least two of the treatment triplicate and integrated for all samples. Finally, total area normalization was performed for the complete set of glycans as well as for the subset of O-GalNAc glycans, to obtain the relative abundancies per glycan in each sample.

### Statistical analyses

Statistical analysis was conducted using the GraphPad Prism software (version Prism 9.5.0). Data are presented as the mean of three independent samples ± standard deviation of the mean (SED) unless otherwise stated. Statistical significance is denoted as * *P* < 0.05, ** *P* < 0.01, *** *P* < 0.001 and **** *P* < 0.0001. IC50, IC70 and IC90 values were determined using GraphPad Prism (version 5.0). For the IC50 a non-linear fit (log (inhibitor) vs. response – variable slope (four parameters)) was made of the data with the least squares fit, the top set at 100 and the bottom between 0 and 10 (due to residual fucoses and sialic acids on the cell membrane). To determine the IC70 and IC90-values, a non-linear fit was made (log(Agonist) vs. response - Find ECanything) with F set at 30 for the IC70 and 10 for the IC90, the top at 100 and the bottom between 0 and 10. Statistics for the lectin panel were determined in GraphPad Prism (version 5.0) using a one-way ANOVA, Dunnett’s multiple comparison tests, with compound vs DMSO (set at 100%).

## Results

### Metabolic fucosylation inhibitors block fucose incorporation in prostate cancer cells and reduce cell proliferation

**Fig. 2:**
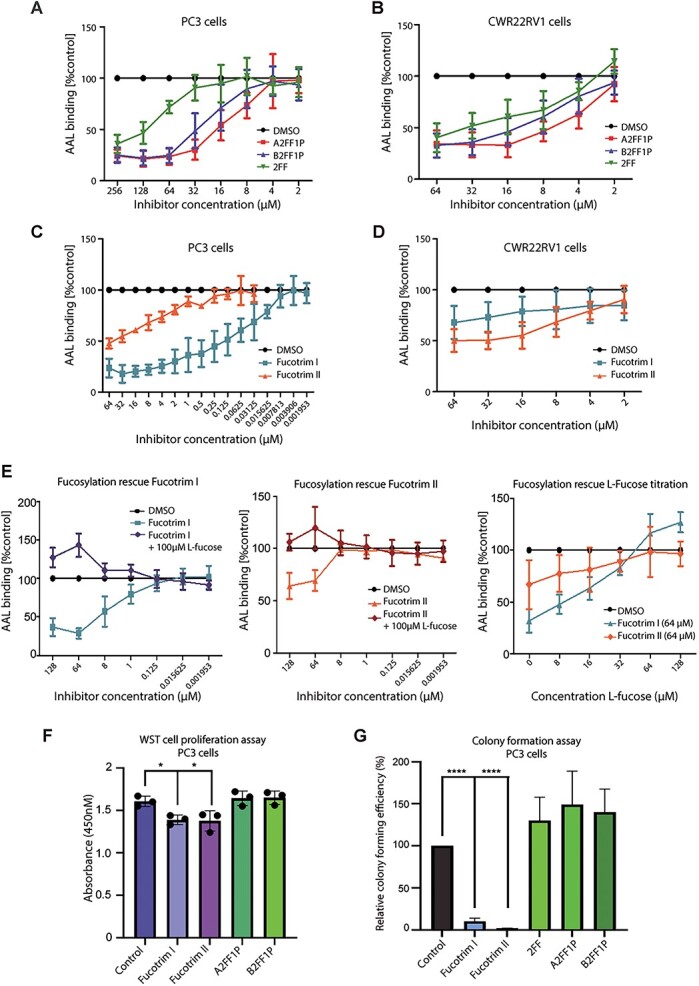
Metabolic fucosylation inhibitors block fucose incorporation in prostate cancer cells and reduce cell proliferation and survival. A) Inhibition of fucosylation in PC3 cells using A2FF1P, B2FF1P & 2FF detected using AAL lectin flow cytometry. Cells were treated with a range of concentrations of each inhibitor from 2 μM to 256 μM for 72 h. The mean fluorescence intensities (MFI) were normalized to a DMSO control. B) Inhibition of fucosylation in CWR22RV1 cells using A2FF1P, B2FF1P & 2FF with a concentration range from 2 to 64 μM for 72 h detected using AAL lectin flow cytometry. The intensities were normalized to a DMSO control. C) Inhibition of fucosylation in PC3 cells using Fucotrim I & Fucotrim II detected using AAL lectin flow cytometry. Cells were treated with a range of concentrations of each inhibitor from 0.001953 μM to 64 μM for 72 h. The intensities were normalized to a DMSO control. D) Inhibition of fucosylation in CWR22RV1 cells using Fucotrim I & Fucotrim II detected using AAL lectin flow cytometry. Cells were treated with a range of concentrations of each inhibitor from 2 μM to 64 μM for 72 h. The intensities were normalized to a DMSO control. For Fig. 1A–D each graph is representative of 3 biological repeats. E) Addition of externally added L-fucose (added in the media in order to enter via the salvage pathway and thereby bypass the de novo inhibition) rescues AAL binding suggesting that the Fucotrim inhibitors act only on the de novo pathway. PC3 cells were treated with a range of concentrations of each inhibitor from 2 μM to 64 μM for 72 h. The intensities were normalized to a DMSO control. (F) WST-1 cell proliferation assays show treatment of PC3 cells with 30 μM Fucotrim I or 60 μM Fucotrim II significantly reduces cell proliferation over 72 h. (G) Treatment with 30 μM Fucotrim I or 60 μM Fucotrim II significantly reduced PC3 cell colony formation over 14 days.

Targeting aberrant fucosylation in prostate cancer represents an exciting opportunity to develop new therapeutic strategies, however, to date this area has been relatively unexplored. Here, we test a panel of metabolic fucosylation inhibitors, including A2FF1P, B2FF1P (Pijnenborg, Visser, et al. 2021), Fucotrim I, and Fucotrim II (Pijnenborg, Rossing, et al. 2021), on prostate cancer cells and monitor the impact on global fucosylation. A2FF1P and B2FF1P are metabolic inhibitors of fucosyltransferases derived from 2-fluorofucose (SGN-2FF). They are metabolically processed via the fucose salvage pathway leading to GDP-2-fluorofucose which is a competitive inhibitor of fucosyltransferases and additionally halts the de novo biosynthesis of GDP-fucose via feedback-inhibition (Fig. 1). Fucotrim I and II are rhamnose-1-phosphate derivatives and are designed to enter the de novo fucose biosynthesis pathway to target the crucial GDP-Mannose 4,6-Dehydratase (GMDS) enzyme (Fig. 1). As Fucotrim I and II are designed to mimic mannose-1-phosphate it has been observed that these compounds also lower the GDP-mannose levels. Using AAL lectin flow cytometry, which predominantly recognizes α-fucose (Bojar et al. 2022), we show that all four fucosylation inhibitors can inhibit fucosylation in both androgen receptor (AR) negative PC3 and AR positive CWR22RV1 prostate cancer cells (Fig. 2A–D), although with different effectiveness, especially for Fucotrim I. See Table 1 for the IC50 and IC70-values (the concentration where a 50% or 70% decrease in lectin binding compared to control was observed). Since often a residual AAL binding of around 30% is found after 3 days of inhibition, the EC70 is considered the concentration around which a maximum effect is seen. We hypothesize that the difference in Fucotrim potency could be due to the fact that Fucotrim I and II affect the de novo biosynthesis of GDP-fucose, therefore cells relying on this route to obtain GDP-fucose might be very sensitive to these inhibitors, but cells that rely more on the salvage pathway to obtain fucose might therefore be less affected by this type of inhibitor. To further validate that the Fucotrim inhibitors act only on the de novo pathway, we conducted a rescue experiment with externally added L-fucose in the media in order to enter via the *salvage* pathway and thereby bypass the de novo inhibition, where we indeed found a complete rescue of AAL binding (Fig. 2E).

We obtained dose-dependent inhibition of fucosylation for both cell lines, and selected concentrations to continue with in further experiments. Next, we investigated the effect of blocking fucosylation on prostate cancer cell proliferation and viability using WST-1, Cell Titer Glo, and colony formation assays. Our findings show that treating PC3 and CWR22RV1 cells with either Fucotrim I or Fucotrim II significantly reduces cell proliferation, viability, and colony formation for both cell lines (Fig. 2F–G and Supplementary Figs 1–3). For SGN-2FF, A2FF1P and B2FF1P, there was no effect on cell proliferation or colony formation. The different effects of these two classes of fucosylation inhibitors might be explained by their distinct mechanisms of action. Since Fucotrim I and II are also found to reduce GDP-mannose levels (Pijnenborg, Rossing, et al. 2021), it could affect more processes than just reducing fucosylation levels as seen with A2FF1P and B2FF1P. We also tested PNT2 and BPH-1 cells, which model normal prostate epithelium and benign prostate hyperplasia respectively, and show that for these cell lines even high concentrations of Fucotrim I and Fucotrim II do not significantly alter cell proliferation rates (Supplementary Fig. 4). Taken together, our data shows that the metabolic fucosylation inhibitors A2FF1P, B2FF1P, Fucotrim I and Fucotrim II can effectively inhibit fucose incorporation into prostate cancer cells, and that treatment with Fucotrim I or Fucotrim II significantly reduces prostate cancer cell proliferation and colony formation.

### Fucotrim I and Fucotrim II downregulate cell cycle gene expression signatures and induce apoptosis in prostate cancer cells

**Fig. 3:**
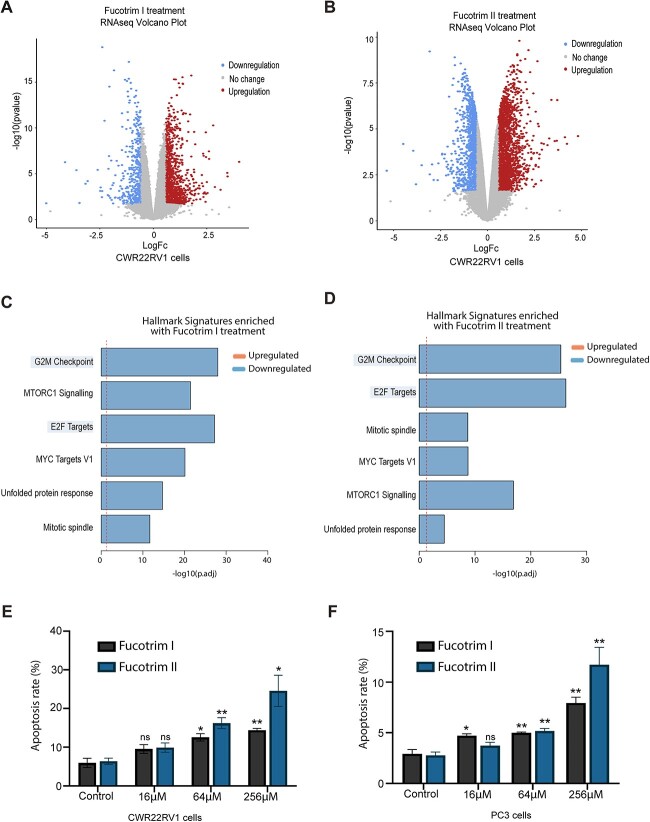
Inhibition of fucosylation in prostate cancer cells using Fucotrim I and Fucotrim II downregulates cell cycle gene expression signatures and induces apoptosis. A) Volcano plot of RNA-seq data to show 1,354 genes that are differentially expressed in CWR22RV1 cells in response to treatment with 30 μM Fucotrim I for 72 h (log2FC > |0.58| + adjusted *P*-value < 0.05). B) Volcano plot to show 3,677 genes that are differentially expressed in CWR22RV1 cells in response to treatment with 60 μM Fucotrim II for 72 h (log2FC > |0.58| + adjusted *P*-value < 0.05). C, D) Ensemble gene set enrichment analysis of genes regulated by Fucotrim I and Fucotrim II reveals downregulation in the “G2M checkpoint” and “E2F targets” hallmark signatures. (E, F) Annexin V flow cytometry shows treatment of CWR22RV1 and PC3 cells with a range of concentrations of Fucotrim I or Fucotrim II induces apoptosis.

To identify signaling pathways controlled by fucosylation inhibitors in prostate cancer, we used RNA-sequencing (RNA-seq) to search for differentially expressed genes upon fucosylation inhibition in prostate cancer cells. Bioinformatic analyses identified 6,222 differentially expressed genes when CWR22RV1 cells were treated with 30 μM Fucotrim I, 7,499 differentially expressed genes when CWR22RV1 cells were treated with 60 μM Fucotrim II, and 2,975 differentially expressed genes when CWR22RV1 cells were treated with SGN-2FF (adjusted *P* value < 0.05) (Fig. 3A and B, Supplementary Fig. 5 and Supplementary Tables 1–4). Hallmark signature enrichment analysis revealed enrichment in 6 gene expression signatures for all three inhibitors, including downregulation in “G2M checkpoint” and “E2F targets” which have both been recently identified as upregulated in the trajectory to prostate tumor progression (Bolis et al. 2021) (Fig. 3C and D and Supplementary Fig. 5). Treatment with FucotrimI/II or SGN-2FF also significantly downregulated the “unfolded protein response (UPR)” gene expression signature (Fig. 3C and D and Supplementary Fig. 5). The UPR is a pro-survival mechanism triggered by endoplasmic reticulum (ER) stress (Madden et al. 2019) that is implicated in the establishment and progression of several cancers including prostate cancer (Storm et al. 2016).

As Fucotrim I and Fucotrim II had such a profound impact on prostate cancer cell proliferation and colony formation, we next investigated if using these inhibitors in prostate cancer cells can promote programmed cell death. Annexin V staining revealed treatment of PC3 and CWR22RV1 cells with either Fucotrim I or Fucotrim II induces apoptosis in both cell lines (Fig. 3E and F and Supplementary Fig. 6). This effect was independent of fucosylation reduction, as apoptosis still occurred when L-fucose was added with the treatment to rescue de novo fucosylation inhibition (Supplementary Fig. 7). Taken together, this data shows that treating prostate cancer cells with Fucotrim inhibitors downregulates gene expression hallmark signatures important in disease progression and induces programmed cell death.

**Table 1:** IC50 and IC70 values for fucosylation inhibitors calculated by AAL lectin flow cytometry.

Compound	IC50 PC3	IC50 CWR22RV1	IC70 PC3	IC70 CWR22RV1
DMSO	N.I	N.I	N.I.	N.I.
A2FF1P	16.26	7.575	29.43	21.15
B2FF1P	26.92	12.86	48.73	31.86
2FF	113.5	25.68	207.0	63.79
Fucotrim I	0.1510	N.D.	0.7560	N.D.
Fucotrim II	29.17	26.96	N.D.	N.D.

N.I = no inhibition. N.D = not determined since the measured values did not reach a reduction to 50% respectively 70% in lectin binding.

### The sialyltransferase inhibitor P-SiaFNEtoc effectively blocks sialylation in prostate cancer cells

**Fig. 4:**
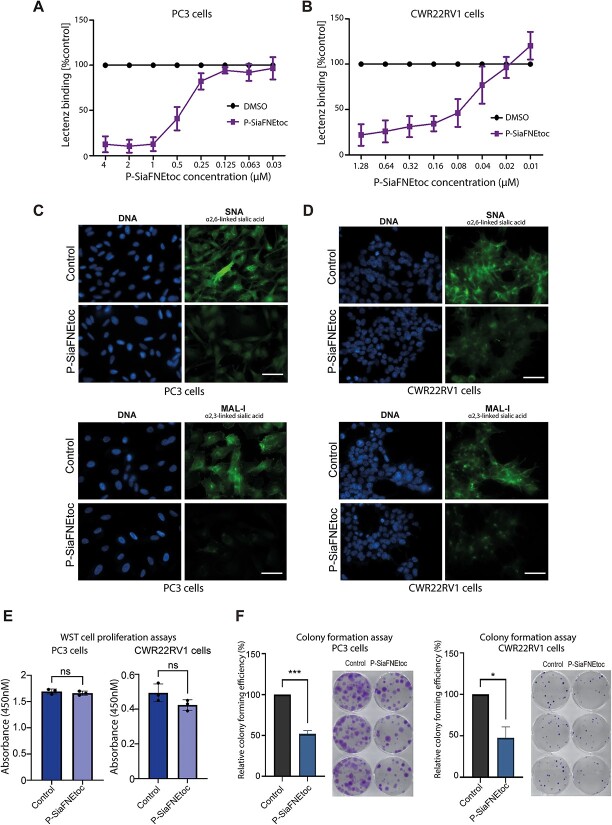
The sialyltransferase inhibitor P-SiaFNEtoc effectively blocks sialylation in prostate cancer cells and reduces colony formation. A, B) Inhibition of sialylation in PC3 and CWR22RV1 cells using P-SiaFNEtoc detected using pan-specific Lectenz lectin flow cytometry (Saunders et al. 2023). Cells were treated with a range of concentrations of P-SiaFNEtoc inhibitor from 0.03 μM to 4 μM for 72 h. The intensities were normalized to a DMSO control. C, D) Detection of sialylated *N*-glycans in prostate cancer cells by immunocytochemistry using SNA and MAL-I lectins. PC3 and CWR22RV1 cells treated with 2 μM P-SiaFNEtoc for 72 h have reduced levels of SNA and MAL-I binding indicating a reduction in both α2-6 and α2-3 linked sialylation in these cells. Scale bar is 20 μm. E) WST-1 cell proliferation assays show treatment of PC3 or CWR22RV1 cells with 2 μM P-SiaFNEtoc hours does not significantly alter cell proliferation over 72 h. F) Treatment of PC3 or CWR22RV1 cells with 2 μM P-SiaFNEtoc significantly reduced colony formation over 14 days. Representative data from 3 biological repeats is shown.

In addition to aberrant fucosylation, alterations to sialylation are also common in prostate cancer and the development of strategies to inhibit sialylated glycans are increasingly being explored as new cancer therapeutics (Munkley 2022). Next, we tested if the previously established C-5-modified 3-fluoro sialic acid sialyltransferase inhibitor, P-SiaFNEtoc (Heise et al. 2019), can inhibit the sialylation of prostate cancer cells in vitro. Using flow cytometry with pan-sialic acid specific Lectenz (Saunders et al. 2023), we obtained dose dependent inhibition of global sialylation for both PC3 and CWR22RV1 cells (Fig. 4A and B). The corresponding IC50 and IC90-values can be found in Table 2. Next, using SNA lectin immunocytochemistry (which recognizes α2-6 linked sialylated *N*-glycans (Bojar et al. 2022)), and MAL-I lectin immunocytochemistry (which recognizes α2-3 linked sialylated glycans (Bojar et al. 2022)), we confirmed inhibition of sialylation in both cell lines following treatment with 2 μM of P-SiaFNEtoc for 72 h (Fig. 4C and D). Although treatment with P-SiaFNEtoc did not impact prostate cancer cell proliferation over 72 h (Fig. 4E), our data suggests sialylation inhibition significantly suppresses the ability of prostate cancer cells to survive and/or to grow in colonies (Fig. 4F). Together, these findings show treatment with low micromolar concentrations of P-SiaFNEtoc effectively blocks sialylation in prostate cancer cells and this significantly reduces the growth of prostate cancer cells in colonies.

### Inhibition of sialylation in prostate cancer cells using P-SiaFNEtoc regulates oncogenic proteins important in prostate tumor progression

**Fig. 5:**
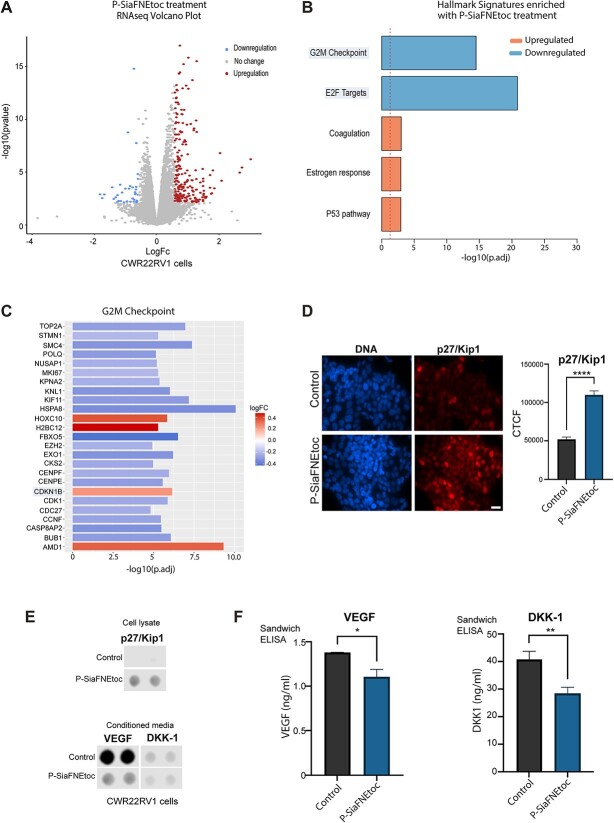
Inhibition of sialylation in prostate cancer cells using P-SiaFNEtoc regulates oncogenic proteins important in prostate tumor progression. A) Volcano plot of RNA-seq data to show 254 genes that are differentially expressed in CWR22RV1 cells in response to treatment with 2 μM P-SiaFNEtoc for 72 h (log2FC > |0.58| + adjusted *P*-value < 0.05). B) Ensemble gene set enrichment analysis of genes regulated by P-SiaFNEtoc treatment reveals downregulation in the “G2M checkpoint” and “E2F targets” hallmark signatures. C) Heatmap to illustrate the top 25 genes with roles in the G2M checkpoint that are significantly differentially expressed in cells treated with P-SiaFNEtoc. D) Validation at the protein level using immunocytochemistry shows p27/Kip1 (which is encoded by the *CDKN1B* gene) is significantly upregulated in CWR22RV1 treated with P-SiaFNEtoc. Scale bar is 20 μm. E) Analysis of CWR22RV1 cells using a proteome profiler oncology Array identified an increase in p27/Kip1 levels in cell lysate samples and a decrease in VEGF and DKK-1 levels in conditioned media samples from cells treated with P-SiaFNEtoc. F) Sandwich ELISA assays confirm significant downregulation of DKK-1 and VEGF in conditioned media samples from P-SiaFNEtoc treated CWR22RV1 cells.

To understand the importance of sialylation in prostate cancer and shed light on the possibility to use sialic acid blockade as a potential therapeutic, we used RNA-seq and an oncology array to identify genes and cancer-related proteins that are differentially expressed upon treatment with P-SiaFNEtoc. Bioinformatic analyses identified 2,302 genes that are differentially expressed in CWR22RV1 cells treated with 2 μM P-SiaFNEtoc (adjusted *P* value < 0.05) (Fig. 5A and Supplementary Table 3) and also identified 5 hallmark signatures that are enriched in P-SiaFNEtoc treated cells (Fig. 5B). These include downregulation of “G2M checkpoint” and “E2F targets” which are known to be upregulated in the progression of prostate tumors (Bolis et al. 2021). Visualization of significantly differentially expressed G2M checkpoint hallmark genes in a heatmap confirmed that P-SiaFNEtoc treatment results in downregulation of G2M checkpoint genes (Fig. 5C). Of particular interest in the G2M checkpoint gene set, the *CDKN1B* gene (which encodes the cyclin-dependent kinase inhibitor 1B (p27/Kip1) (Chu et al. 2008)), was significantly upregulated (adjusted *P* value < 0.0001, Log_2_FC 0.24). p27/Kip1 is a negative regulator of the cell cycle, and numerous immunohistochemical studies have shown that low p27/Kip1 expression in prostate cancer tissue is a predictive factor for disease recurrence after radical prostatectomy (Cote et al. 1998; Tsihlias et al. 1998; Yang et al. 1998; Vis et al. 2000, 2002). Validation at the protein level using immunocytochemistry confirmed that P-SiaFNEtoc treatment promotes an increase of p27/Kip1 in CWR22RV1 cells (Fig. 5D and E). Next, using an oncology array, we analyzed expression of 84 oncogenic proteins in cell lysates and conditioned media samples from prostate cancer cells treated with P-SiaFNEtoc. This confirmed upregulation of p27/Kip1 in CWR22RV1 cells and identified 13 proteins that are potentially reduced in the secretome of prostate cancer cells following sialic acid blockade (Supplementary Fig. 8). A particularly strong reduction in secreted Vascular Endothelial Growth Factor (VEGF) (a key mediator of angiogenesis and a target in cancer therapy (Carmeliet 2005)) was observed (Fig. 5E). Further validation using sandwich ELISA assays confirmed significant downregulation of VEGF, and significant downregulation of the secreted Wnt inhibitor Dickkopf-1 (DKK-1), which is upregulated in the serum of prostate cancer patients and linked to poor survival (Rachner et al. 2014), in conditioned media samples from CWR22RV1 cells treated with P-SiaFNEtoc (Fig. 5F). In summary, these results highlight the important role of sialylation in prostate tumor pathology and reveal the potential to exploit the sialylation inhibitor P-SiaFNEtoc, once improved to be more targeted, as a novel treatment for prostate cancer.

### Dual and specific inhibition of sialylation and fucosylation in prostate cancer cells

**Fig. 6:**
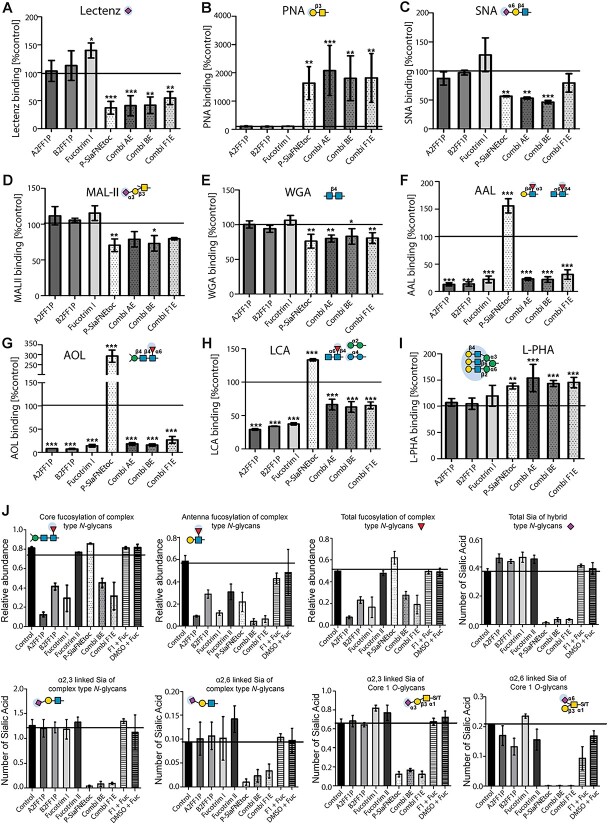
Dual inhibition of sialylation and fucosylation in prostate cancer cells does not promote any side effects on other glycans. Glycome analysis of PC3 prostate cancer cells treated with specific mono- or combination treatments targeting fucosylation and sialylation using a lectin panel. PC3 cells were treated with 100 μM A2FF1P, 100 μM B2FF1P, 30 μM Fucotrim I, 2 μM P-SiaFNEtoc, or with the combination therapies AE (A2FF1P + P-SiaFNEtoc), BE (B2FF1P + P-SiaFNEtoc), and F1E (Fucotrim1 + P-SiaFNEtoc). Lectin flow cytometry was performed for A) pan-specific Lectenz, B) PNA lectin, C) SNA lectin, D) MAL-II lectin, E) WGA lectin, F) AAL lectin, G) AOL lectin, H) LCA lectin, and I) L-PHA lectin. Signal intensities were compared to a DMSO only control. J) *N*-glycan and *O*-glycan mass spectrometry analysis of inhibitor and control treated PC3 cells. Monosaccharide symbols follow the SNFG (Symbol Nomenclature for Glycans) system (Varki et al. 2015).

It has been reported that fucosyltransferase and sialyltransferase enzymes can compete for the same acceptor substrates, meaning that selective inhibition of sialylation can lead to increased levels of overall fucosylation and vice versa (Beyer et al. 1979; Rillahan et al. 2012; Mondal et al. 2015). Therefore, due to the important role both fucosylation and sialylation play in cancer progression, it could be unfavorable to inhibit either sugar alone. To explore this potential issue, and identify potential global effects of our inhibitors on other glycosylation pathways, we treated PC3 prostate cancer cells with the panel of fucosylation and sialylation inhibitors alone or in combination and measured the effect of these treatments on the glycome using a lectin panel. Single treatment with P-SiaFNEtoc reduces global sialylation (detected using the pan-specific Lectenz (Saunders et al. 2023)) and increases binding of PNA lectin, which recognizes uncapped galactose (Bojar et al. 2022) (Fig. 6A and B). P-SiaFNEtoc reduced binding of SNA lectin and MAL-II lectin (that recognize α2–6 linked and α2-3 linked sialic acid respectively (Bojar et al. 2022)) (Fig. 6C and D) and also significantly reduced binding of WGA lectin (which recognizes mainly chitobiose (GlcNAc), but which can also interact with sialic acid containing glycans (Ryva et al. 2019) (Fig. 6E).

Single treatment with either of the fucosylation inhibitors A2FF1P, B2FF1P, and Fucotrim I strongly reduced binding of AAL and AOL lectins, indicating a reduction in global fucosylation (Fig. 6F and G), and also reduced binding of LCA lectin indicating a reduction in core fucosylation (Bojar et al. 2022) (Fig. 6H). Furthermore, our data shows that inhibiting sialylation or fucosylation did not gravely impact *N*- glycan branching, recognized by L-PHA lectin which recognizes the β1,6-branch of the *N*-glycan backbone (Bojar et al. 2022) (Fig. 6I), although lectin binding is slightly higher with PC3 cells treated with P-SiaFNEtoc, likely because α2,6-linked sialic acid can mask the binding epitope of this lectin (Gao et al. 2019). Consistent with previous studies, we detected an increase in sialic acid levels following inhibition of fucosylation using Fucotrim I (Fig. 6A), and an increase in fucosylation after treatment with P-SiaFNEtoc (Fig. 6F–H), likely because the terminal sites on glycans occupied by fucose had now become available for sialic acid incorporation and vice versa. Another possible explanation is that removal of a fucose positively influences binding of the lectin recognizing sialic acids and vice versa. Combination treatment with both fucosylation and sialylation inhibition led to the specific reduction in both sialic acid (Fig. 6A) and fucose containing glycans (Fig. 6F–H). We also treated CWR22RV1 prostate cancer cells with our inhibitor panel where we obtained similar results using our lectin panel (Supplementary Fig. 9).

To assess if a reduction of GDP-Mannose in Fucotrim treated cells could lead to *N*-glycosylation alterations in the mannosylation step and be the potential cause for the effect seen on cell death, we compared the effect on overall lectin binding of the Fucotrim inhibitors with 2-Deoxy-Glucose (2-DG), a compound known to affect *N*-glyosylation in the mannosylation step which can be reversed by addition of extracellular D-Mannose (Kurtoglu et al. 2007). We found that binding of lectin L-PHA (*N*-glycan branching) was reduced by 2-DG and that this was indeed reversible by addition of extracellular D-Mannose, a feature not shared with Fucotrim treated cells (Supplementary Fig. 10).

**Table 2:** IC50 and IC90 values for sialylation inhibition calculated using Lectenz flow cytometry.

Compound	IC50 PC3	IC50 CWR22RV1	IC90 PC3	IC90 CWR22RV1
DMSO	N.I	N.I	N.I.	N.I.
P-Siafnetoc	0.3964	0.08700	0.8363	0.4490

N.I = no inhibition.

To further probe the effects of the inhibitors on *N*-glycan and *O*-glycan biosynthesis, we performed *N*-glycan and *O*-glycan mass spectrometry analysis of inhibitor and control treated PC3 cells (Fig. 6J and Supplementary Figs 11–12). In line with the lectin binding experiments, reduced *N*-glycan fucosylation was observed after A2FF1P, B2FF1P and Fucotrim I treatment. The effects of these inhibitors on core fucosylation and antenna fucosylation were comparable and the addition of L-fucose restored the fucosylation in Fucotrim I treated cells. No fucosylation of *O*-glycans was found on the PC3 cells in either control or treatment conditions. Fucotrim II showed a slight reduction in *N*-glycan antenna fucosylation, while core fucosylation was unaffected. Notably, no effect on *N*-glycan mannosylation was detected in PC3 cells treated with the Fucotrim compounds, nor was the inhibitor found to be built into the glycan structures. In P-SiaFNEtoc treated cells, sialylation was almost completely depleted (both for *N*-glycan and Core 1 *O*-GalNAc-glycans) with no apparent differential effect on α2-3 and α2-6 linked sialic acids. The increase in AAL and AOL lectin binding after P-SiaFNEtoc treatment was only partly reflected by a small increase in total fucosylated *N*-glycan structures (Fig. 6J), while specifically antenna fucosylation was slightly decreased. Vice versa, fucosylation inhibitor treatment resulted in a slight increase of sialic acid capping on hybrid type *N*-glycans. The combined treatment of two inhibitors resulted in loss of both sialylated and fucosylated glycans without side-effects on other glycosylation features. Another finding, not investigated by the lectin panel, was a decrease in Tn antigen for Fucotrim I and P-SiaFNEtoc treated cells (Supplementary Fig. 12). Interestingly, we found the incorporation of 2FF1P derived inhibitors in the *N*-glycan data, showing between 0.8 and 1.1% of the total glycan species to contain 2FF1P fucosylation. This was not observed for the other tested inhibitors. Taken together, these data show that both the fucosylation and sialylation inhibitors tested are very specific for their respective family of glycosyltransferases and respective metabolic biosynthesis pathways with minor apparent side effects on other glycan structures, even with dual inhibitor treatment. Furthermore, our findings suggest that parallel and specific inhibition of sialylation and fucosylation can be achieved with inhibitor combinations allowing studies into their dual effects in cancer biology and competing biosynthetic routes.

## Discussion

Aberrant glycosylation is a hallmark of cancer (Munkley and Elliott 2016) and new treatment options targeting tumor-associated glycans and their biosynthesis are currently being tested in several clinical trials (Mereiter et al. 2019; Munkley 2022). Two of the most common changes in the glycome of cancer cells are aberrant sialylation (addition of sialic acid to glycans) and fucosylation (addition of fucose to glycans) (Pinho and Reis 2015). Inhibitors targeting both fucose (SGN-2FF) and sialic acid (P-3F_AX_-Neu5Ac) have been developed and have shown promising results as cancer therapeutics. However, P-3F_AX_-Neu5Ac was found to induce renal toxicity in mice (Macauley et al. 2014), and while a clinical trial with SGN-2FF produced a significant drop in tumor burden, the study was terminated due to safety concerns (NCT 02952989) (Do et al. 2021). The efficacy of P-3F_AX_-Neu5Ac and SGN-2FF, and the potential to inhibit formation of sialylated and fucosylated glycans to block tumor growth, metastasis and immune evasion, has inspired the development of new potent metabolic inhibitors that induce inhibition of sialic acid or fucose incorporation. These inhibitors reach higher effective concentrations within the cell thereby increasing their potency and rendering them good tool compounds to study the effect of global metabolic inhibition of sialylation and fucosylation in cancer cells.

Here, we explore the potential of a panel of potent metabolic inhibitors of fucosylation and sialylation in prostate cancer cell models. Our findings identify a panel of inhibitors that act to block the synthesis of sialylated and fucosylated glycan epitopes within days without significantly altering other glycan structures. Furthermore, we reveal that treatment of PC3 and CWR22RV1 prostate cancer cells with the fucosylation inhibitors Fucotrim I or Fucotrim II induces apoptosis. As the fucosylation inhibitors A2FF1P and B2FF1P do not appear to alter cell proliferation or colony formation, and Fucotrim II only shows a slight effect on fucosylation, our findings suggest that the impact of Fucotrim I/II on prostate cancer cells is likely to not only be attributed to fucosylation inhibition. Since the Fucotrim inhibitors resemble mannose and have been shown to reduce both GDP-fucose and GDP-mannose levels, this might be an alternative mechanism by which apoptosis is induced, however our lectin panel data and mass spectrometry glycan profiling did not provide further insights into this. Although we found no differences in *N*-glycan mannosylation, we did not investigate the effects on total *N*-glycan abundance or C-mannosylation which may be relevant. Alternatively, a reduction in proliferation has also been reported for fucose-derivatives which are fluorinated at the 6-position, similar to the Fucotrims (Dai et al. 2020). These 6-fluorofucose inhibitors were reported to be more anti-proliferative, but less potent inhibitors of FUT8-mediated fucosylation compared to 2FF. It was hypothesized that these compounds might hit additional targets other than FUT8 which may cause these anti-proliferative effects. As the 6-fluorination feature is shared between the two molecules classes as well as their anti-proliferative effects, it would be interesting to investigate the mechanism behind Fucotrim-induced apoptosis further. We also show that sialic acid blockade with the sialyltransferase inhibitor P-SiaFNEtoc downregulates the expression of genes and proteins important in prostate cancer progression. It remains to be determined whether the reduced expression of cancer-related proteins in response to sialylation inhibition results from the reduced secretion of individual cancer-related glycoproteins or a global effect on gene expression and the secretory pathway.

A recent study profiled the trajectory of prostate cancer and revealed most cancers follow a uniform roadmap to tumor progression characterized by upregulation of cell cycle progression, including increased expression of G2M checkpoints (Bolis et al. 2021). Here, we find that inhibition of sialylation in prostate cancer cells significantly downregulates G2M checkpoint gene expression patterns and increases levels of the cell cycle inhibitory protein p27/Kip1. Therapies focused on targeting and inactivating the G2M checkpoint are a common anti-cancer strategy and many compounds have been developed that specifically inhibit the G2M transition (Kawabe 2004; Bucher and Britten 2008). Reduced expression of the tumor suppressor p27/Kip1 has been reported as an independent prognostic factor for poor clinical outcome in a range of tumor types, including breast, colon and lung (Lloyd et al. 1999). As loss of p27/Kip1 in human cancer is frequently due to increased proteasomal degradation rather than mutations in *CDKN1B*, therapies that can restore and stabilize p27/Kip1 are of interest (Nickeleit et al. 2007; Rico-Bautista et al. 2010). The finding that P-SiaFNEtoc can increase p27/Kip1 expression in prostate cancer cells in vitro warrants further exploration in other cancer cell lines with low endogenous p27/Kip1. Our findings suggest that treatment with Fucotrim I/II or SGN-2FF also downregulates UPR gene expression signatures, indicating that inhibiting fucosylation can have profound effects on processes such as the UPR in prostate cancer cells. Targeting the adaptive survival aspects of the UPR has been highlighted as a promising approach for prostate cancer therapy (Storm et al. 2016) pointing to the need for future studies to further understand the cytotoxic effects of Fucotrim I/II in prostate cancer cells and further investigation into how this impacts the UPR.

Our study also shows that sialic acid blockade can significantly suppress the secretion of both VEGF and DKK-1 by prostate cancer cells. The pro-angiogenic factor VEGF is upregulated in aggressive prostate cancer, is linked to metastasis, and therapies targeting VEGF pathways are being investigated in clinical trials (Botelho et al. 2010; Roberts et al. 2013). DKK-1 is a potent inhibitor of the Wnt signaling pathway that can modulate immune cell activities, promote tumor growth in mouse models of prostate cancer, and is a promising target for cancer immunotherapy (Thudi et al. 2011; Chu et al. 2021). Our finding that P-SiaFNEtoc treatment downregulates the G2M checkpoint hallmark pathway, increases expression of p27/Kip1 and inhibits VEGF and DKK-1 proteins, suggests that sialylation inhibition targets key proteins critical to prostate cancer disease progression, and has important and wide-ranging implications for the treatment of prostate cancer patients.

The aberrant glycosylation of tumors can contribute to therapy resistance in cancer (Munkley 2022), highlighting that targeting glycans in combination with existing therapies could boost the response to treatment in prostate cancer patients. Of particular interest, immunotherapy strategies can likely be combined with therapies targeting glycosylation. Sialic acid blockade in mouse tumor models boosted tumor eradication by antigen-specific effector T cells immunity (Büll et al. 2018). Immune checkpoint blockade has revolutionized treatment for many patients with cancer, but despite success against other cancers, prostate tumors have so far resisted immunotherapy (King 2022). Thus, strategies to enhance immunotherapy are essential to broaden the population of prostate cancer patients who may benefit. Studies suggest that the anti-tumor activity of SGN-2FF is immune dependent (Alley et al. 2017) and a clinical trial for advanced solid tumors tested inhibition of fucosylation as a monotherapy and in combination with immune checkpoint blockade (SGN-2FF combined with the PD-1 inhibitor pembrolizumab) (NCT 02952989) (Do et al. 2021). Similarly, increased sialylation of tumors is a well-established driver of immune escape and targeting hypersialylation can improve anti-tumor responses (Munkley 2022). Thus, inhibition of sialylation is likely an effective approach to augment immunotherapy and a combination therapy combining desialylation and immune checkpoint blockade is currently in clinical trials for other cancer types (NCT05259696) (Gray et al. 2020; Stanczak et al. 2022). Interestingly, M2 macrophage polarization is closely linked to prostate cancer progression (Bolis et al. 2021), and inhibiting sialylation has been shown to repolarize tumor-associated macrophages to enable effective immune checkpoint blockade (Stanczak et al. 2022). Therefore, it is interesting to speculate that combining potent metabolic inhibitors of fucosylation and sialylation with immunotherapy will lead to the development of new combination therapies for prostate cancer.

## Conclusion

In conclusion, our study identifies a panel of potent metabolic inhibitors with the potential to effectively target aberrant fucosylation and sialylation in prostate cancer cells without altering other glycan structures. Furthermore, we demonstrate that blocking fucose and sialic acid incorporation into prostate cancer cells inhibits genes and proteins important in prostate cancer progression. Our study highlights the future potential to target aberrant glycosylation, both as a monotherapy and in possible combination with immune checkpoint blockade, to develop new treatments for prostate cancer. Moving forward, we envisage our study will lead to the broader use of metabolic inhibitors to explore the role of fucosylated and sialylated glycans in prostate cancer, and that in vivo studies using these inhibitors will further investigate the potential to block fucose and sialic acid incorporation to inhibit prostate tumor progression-paving the way for the development of a new class of prostate cancer therapeutics.

## Supplementary Material

Supplementary_Figures_cwad085

Supplementary_Tables_I-IV_cwad085

## Data Availability

The authors confirm that the data supporting the findings of this study are available within the article and its Supplementary Materials.
