# Supplementary material for: Targeting aberrant sialylation and fucosylation in prostate cancer cells using potent metabolic inhibitors
Source: Glycobiology. 2023 Oct 17;33(12):1155–71. doi: 10.1093/glycob/cwad085 (PMC10876042; doi:10.1093/glycob/cwad085)

Supplementary Figure 1. WST-1 proliferation and Cell Titer Glo cell viability assays for PC3 and CWR22RV1 cells treated with the fucosylation inhibitors Fucotrim I and Fucotrim II. (**A**) WST-1 cell proliferation assays show treatment of CWR22RV1 cells with 10 μM and 30 μM Fucotrim I, or 50 μM, 100 μM, and 150 μM of Fucotrim II significantly reduces cell proliferation over 72 hours. (**B,C**) CellTiter-Glo luminescent assays show a dose response in cell viability for both PC3 and CWR22RV1 cells treated with both inhibitors.

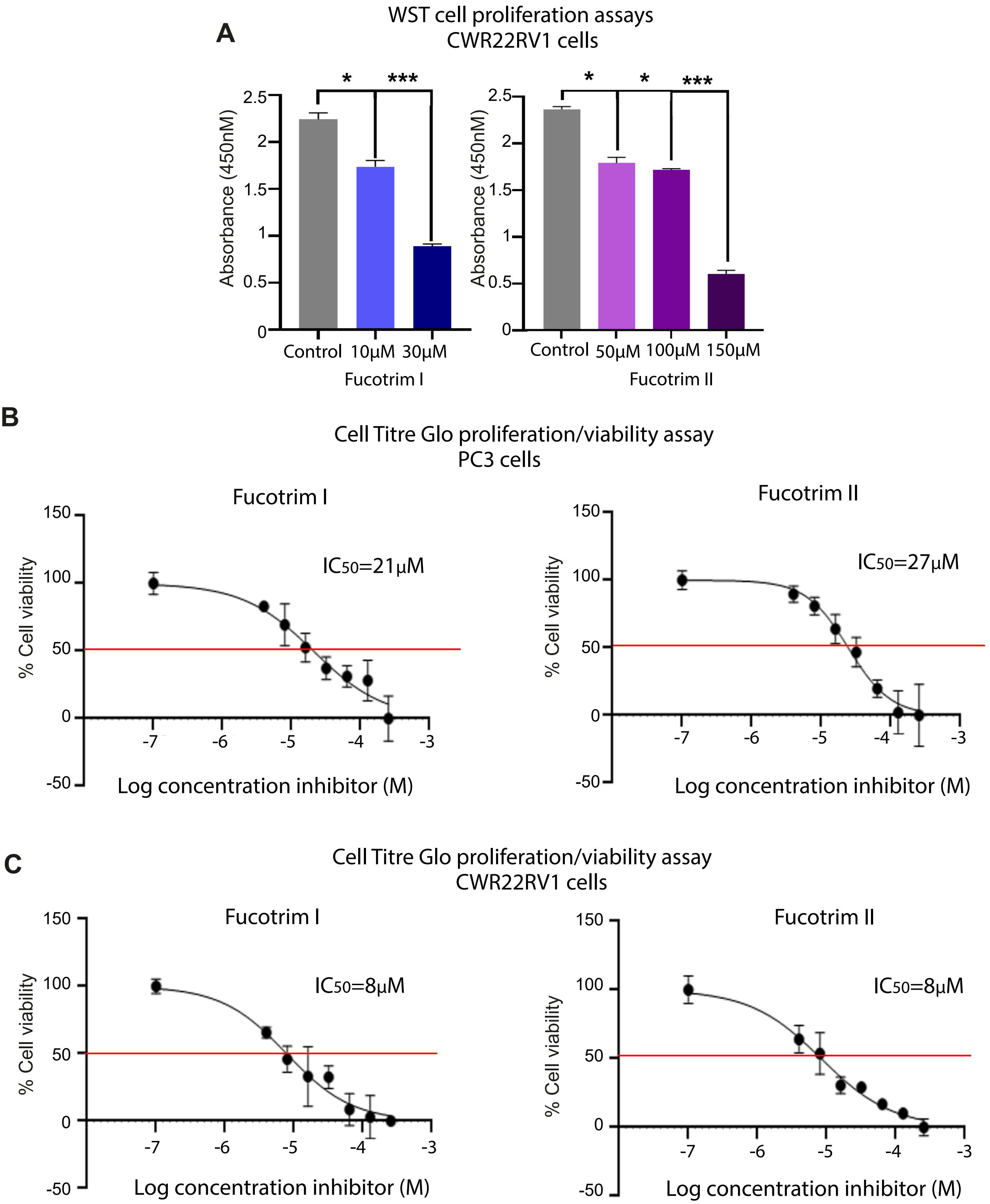

Supplementary Figure 2. Representative images from PC3 colony formation assays in Figure 2G.

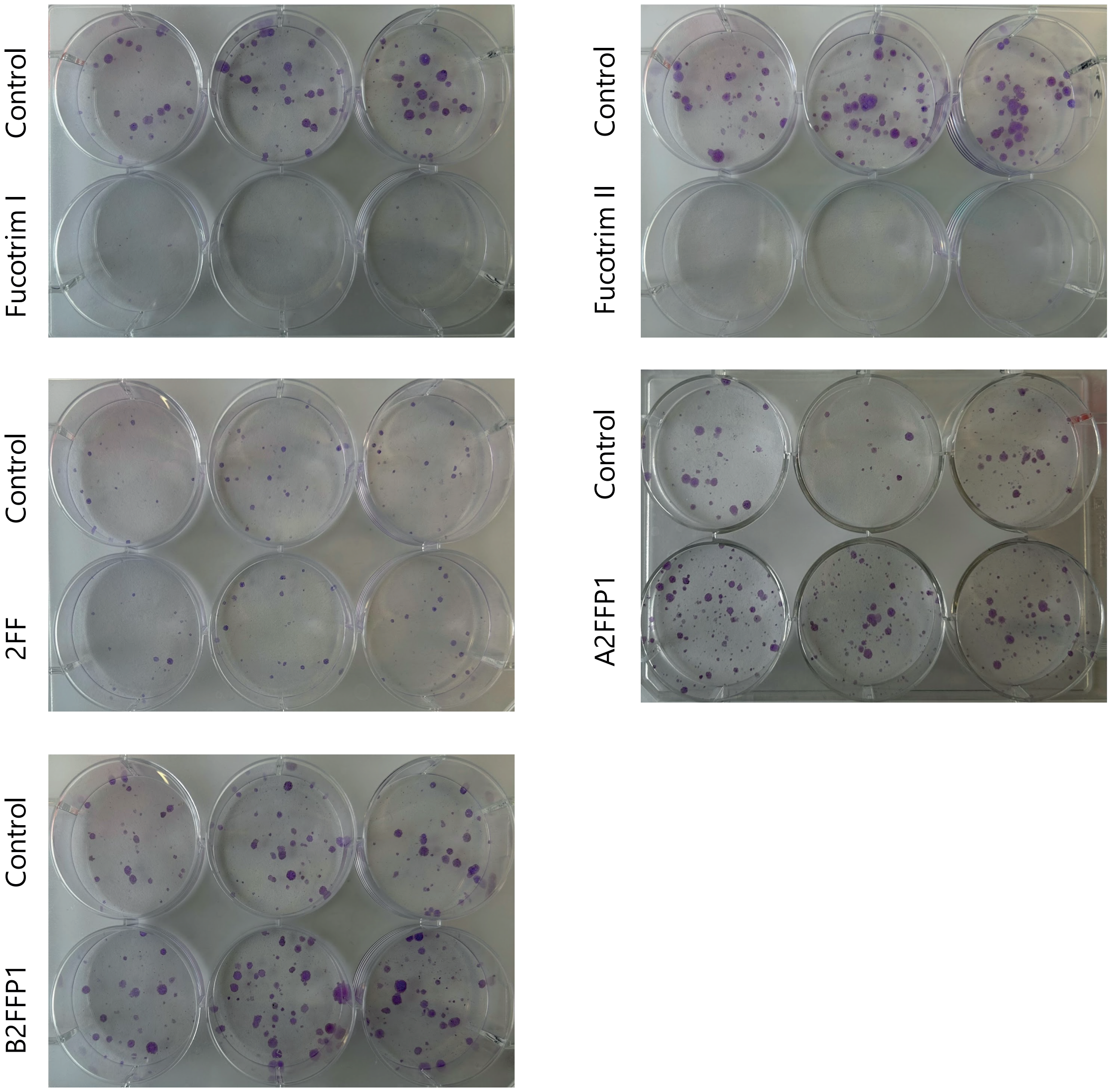

Supplementary Figure 3. CWR22RV1 cells colony formation assays following treatment with fucosylation inhibitors for 72 hours.

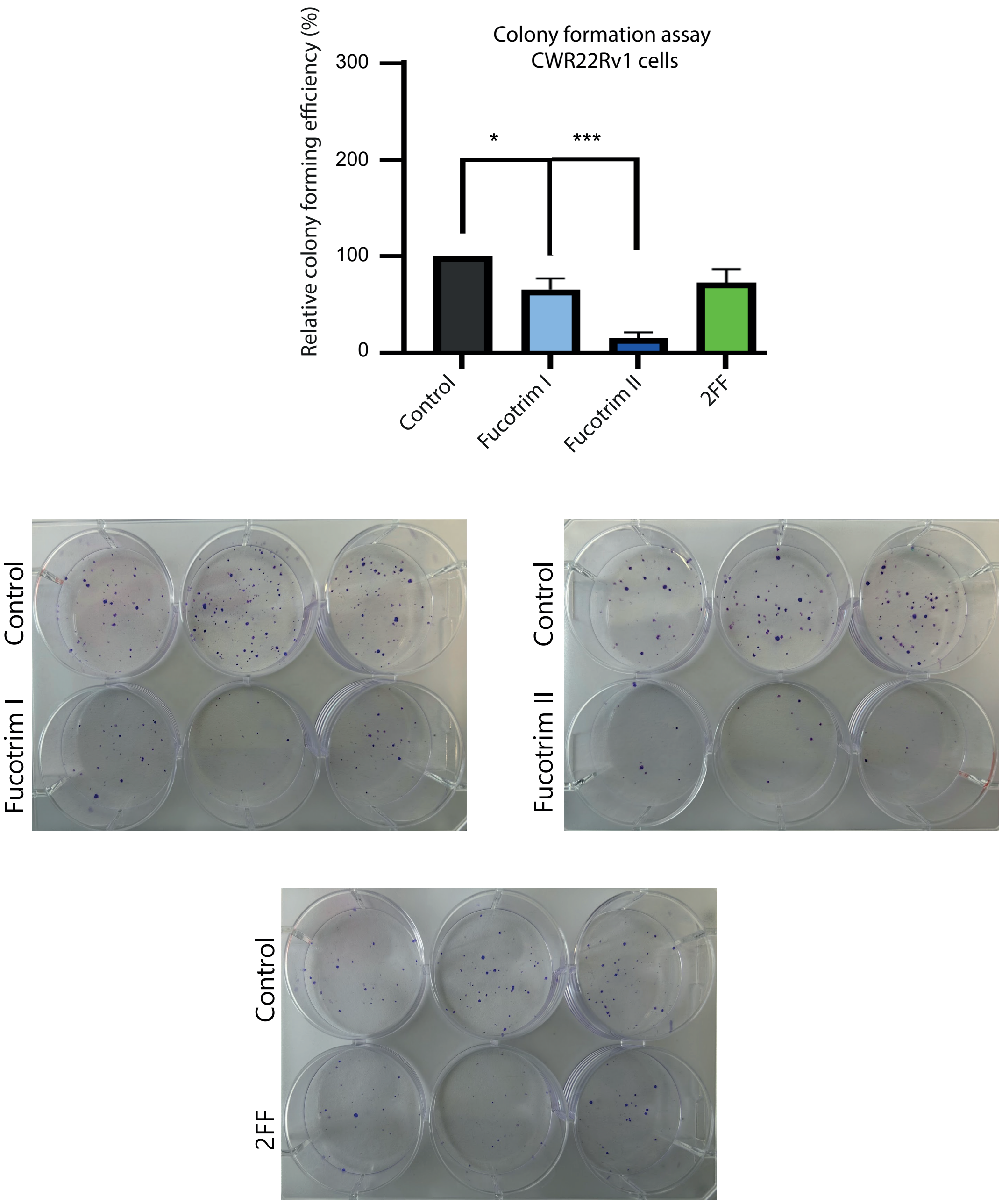

Supplementary Figure 4. Fucotrim I and Fucotrim II do not impact the proliferation rates of normal or benign prostate cancer cell lines. (**A**) WST-1 proliferation assays for PNT2 normal prostate cells. (**B**) WST-1 proliferation assays for BPH-1 benign prostate cells.

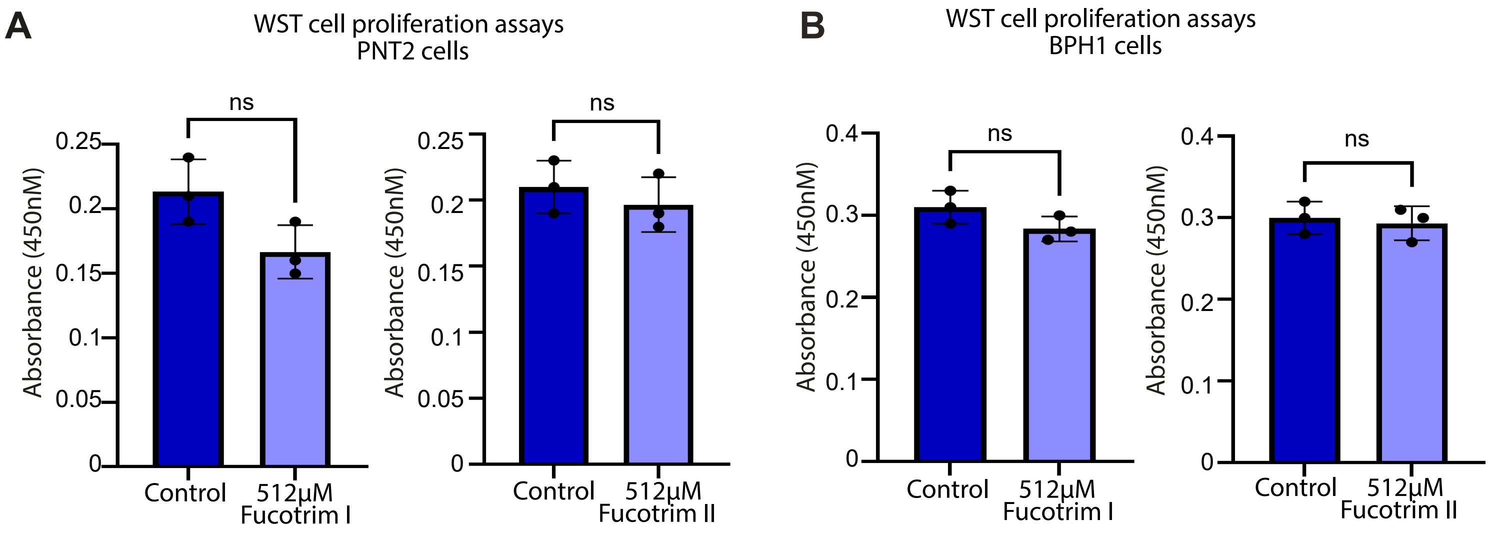

Supplementary Figure 5. (**A**) Volcano plot of RNA-seq data to show 2957 genes that are differentially expressed in CWR22RV1 cells in response to treatment with 100 µM SGN-2FF for 72 hours (log2FC > |0.58| + adjusted p-value <0.05). (**B**) Ensemble Gene Set Enrichment Analysis of genes regulated by SGN-2FF reveals downregulation in the 'G2M checkpoint', 'E2F targets', and 'UPR' hallmark signatures.

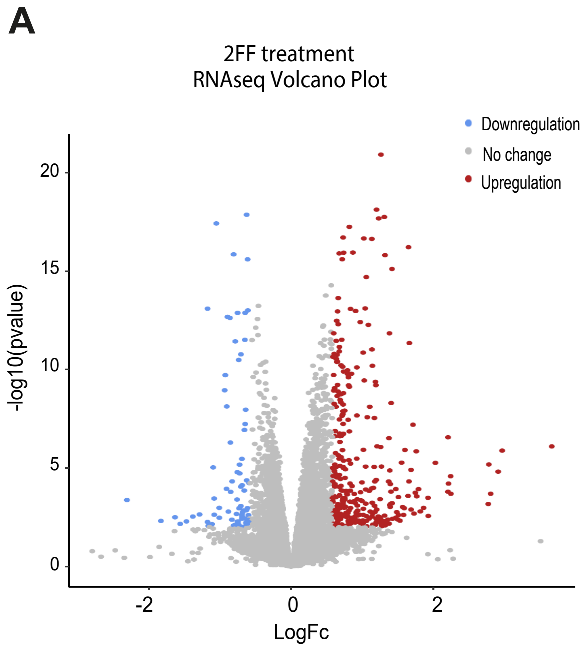

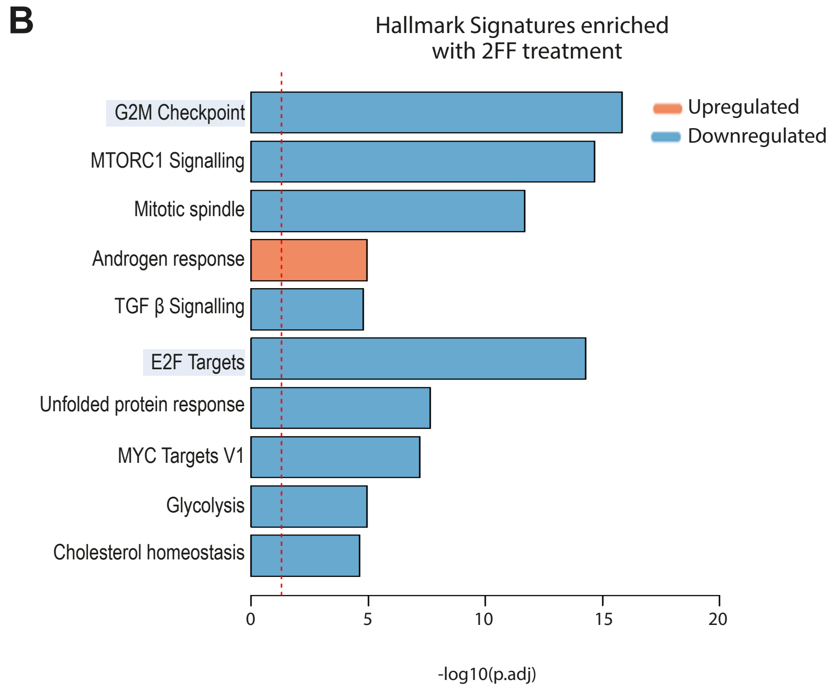

Supplementary Figure 6. Representative experiments depicting the 4 quadrants for the Annexin staining shown in Figures 3E and 3F.

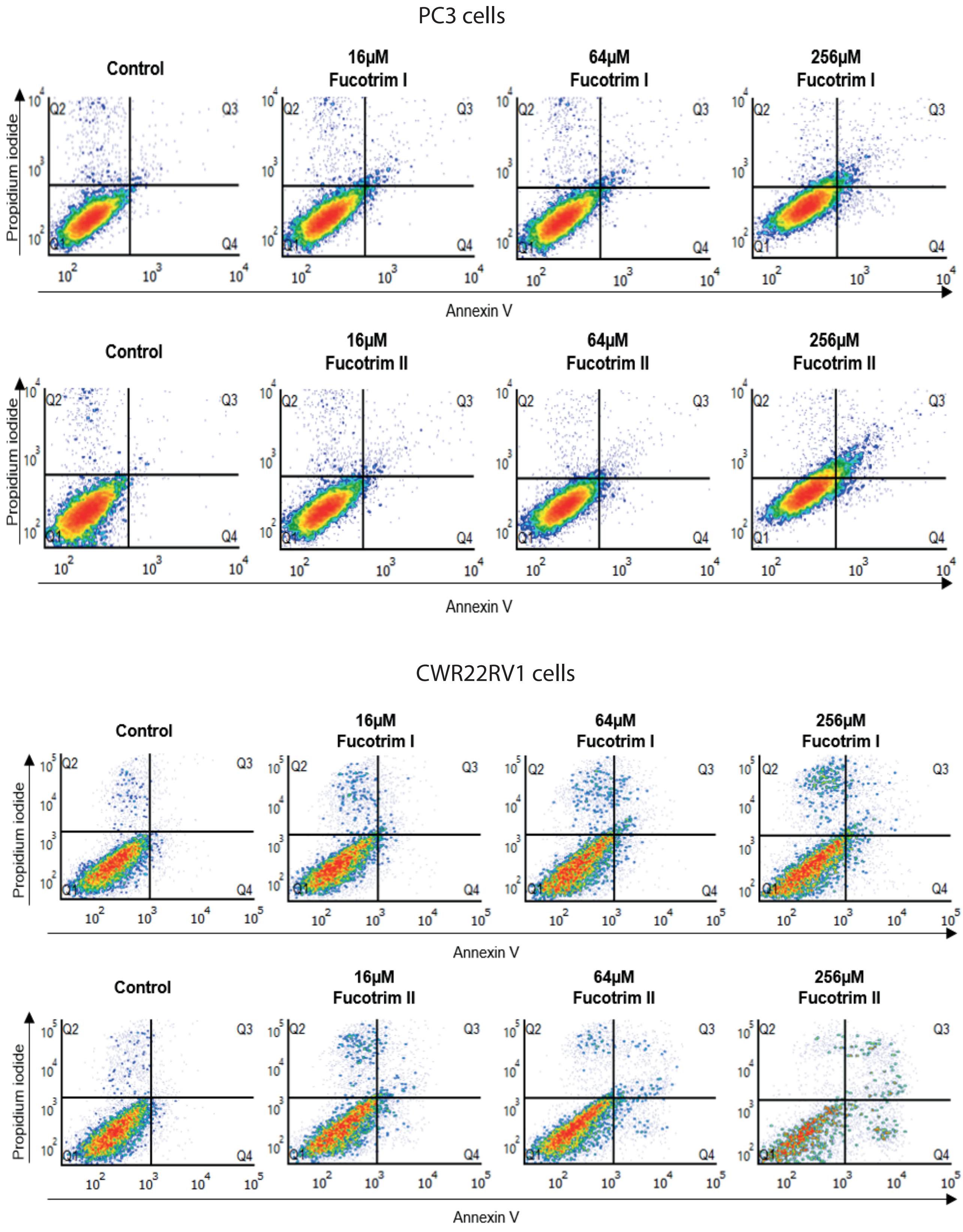

Supplementary Figure 7. Fucose rescue experiment apoptosis assay. PC3 cells were treated with 64 μM Fucotrim I or 64 μM Fucotrim II with and without L-fucose added to the cell culture media. After 72 hours inhibitor treatment alone significantly induced apoptosis. No significant effect on apoptosis was detected when L-fucose was also included in the media compared to treatment alone.

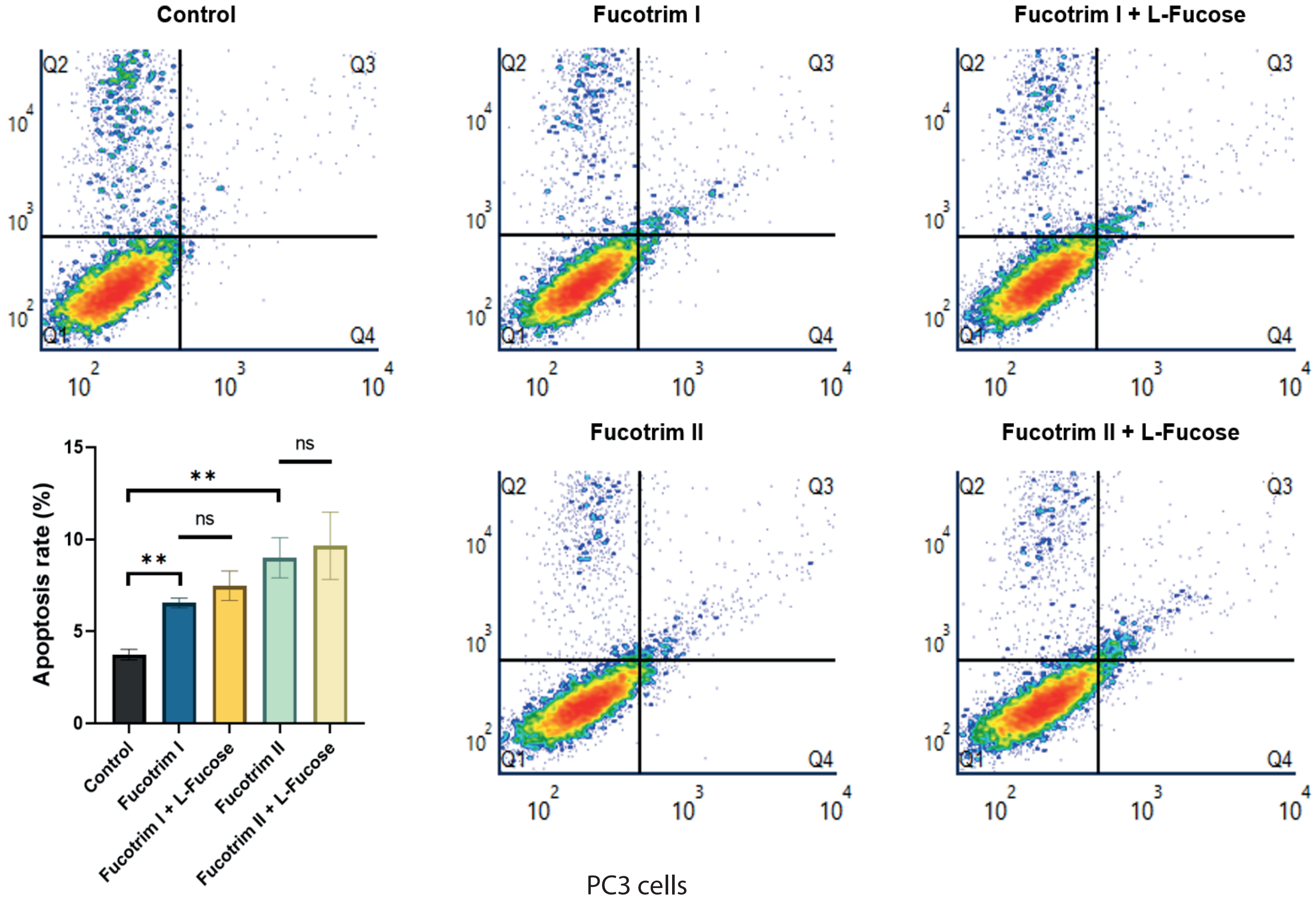

Supplementary Figure 8. Oncology array analysis of (**A**) cell lysates (proteome) and (**B**) conditioned media (secretome) samples from CWR22RV1 cells treated with 2 µM P-SiaFNEtoc.

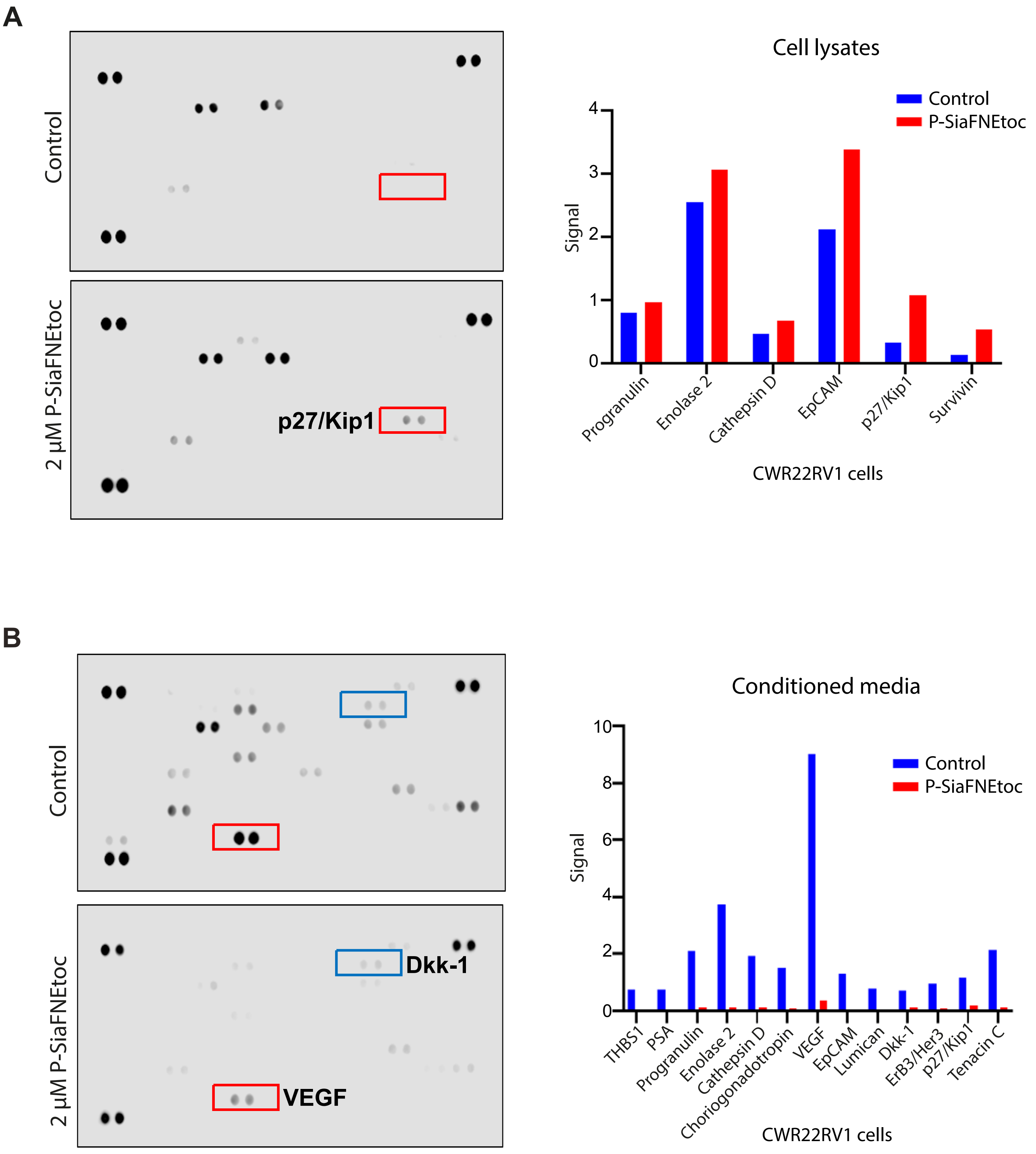

Supplementary Figure 9. Dual inhibition of sialylation and fucosylation in CWR22RV1 prostate cancer cells. Glycome analysis of CWR22RV1 prostate cancer cells treated with specific mono- or combination treatments targeting fucosylation and sialylation using a lectin panelCWR22RV1 cells were treated with 100 µM A2FF1P, 100 µM B2FF1P, 30 µM Fucotrim I, 2 µM P-SiaFNEtoc, or with the combination therapies AE (A2FF1P + P-SiaFNEtoc), BE (B2FF1P + P-SiaFNEtoc), and F1E (Fucotrim1 + P-SiaFNEtoc). Lectin flow cytometry was performed for (**A**) pan-specific Lectenz, (**B**) PNA lectin, (**C**) SNA lectin, (**D**) MAL-II lectin, (**E**) AAL lectin, (**F**) AOL lectin, (**G**) LCA lectin, (**H**) WGA lectin, and (**I**) L-PHA lectin. Signal intensities were compared to a DMSO only control. Monosaccharide symbols follow the SNFG (Symbol Nomenclature for Glycans) system (Varki et al. 2015).

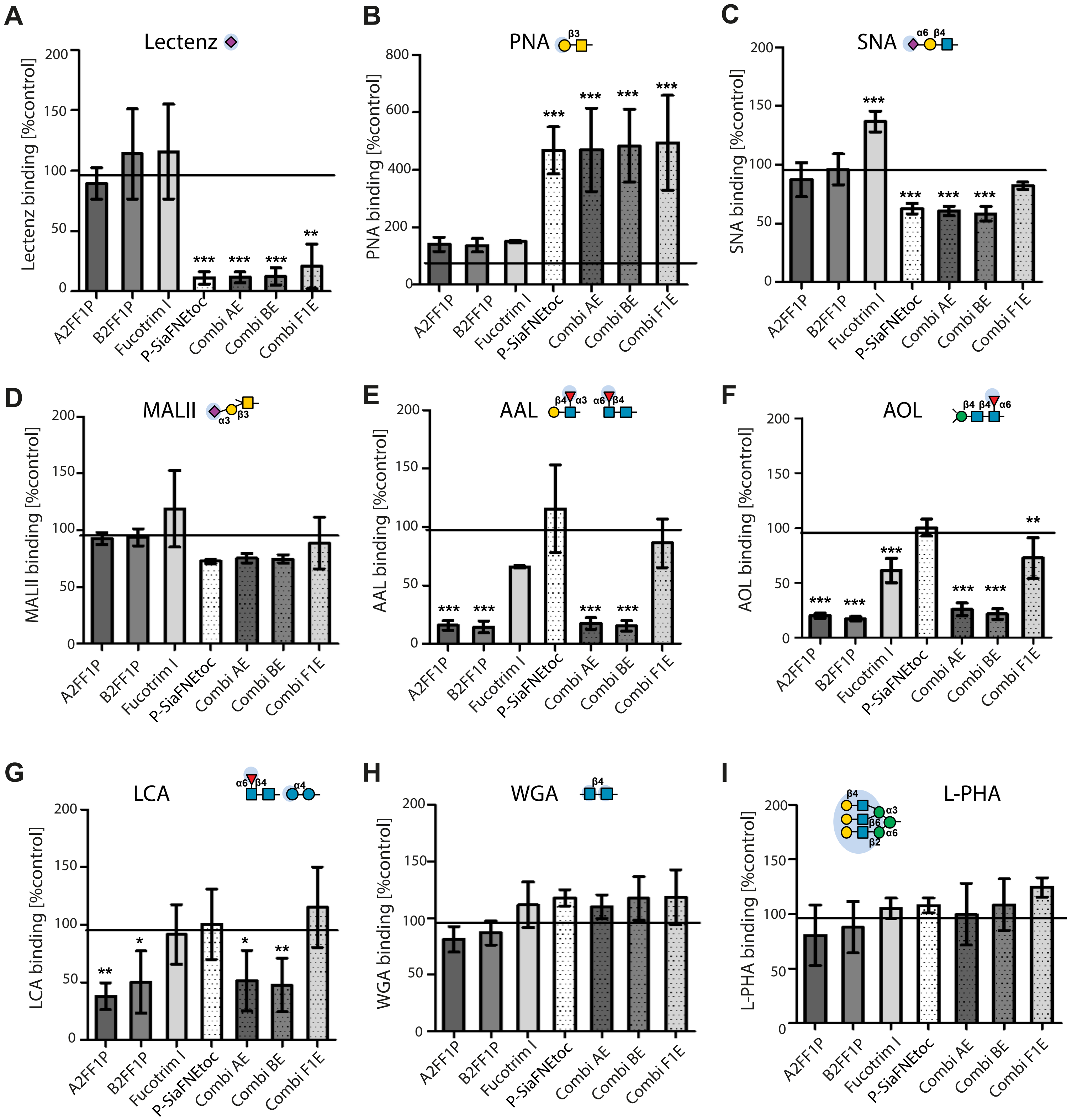

Supplementary Figure 10. (**A**) Lectin panel for PC3 cells treated with 2nM 2DG. (**B**) D- mannose rescue experiment.

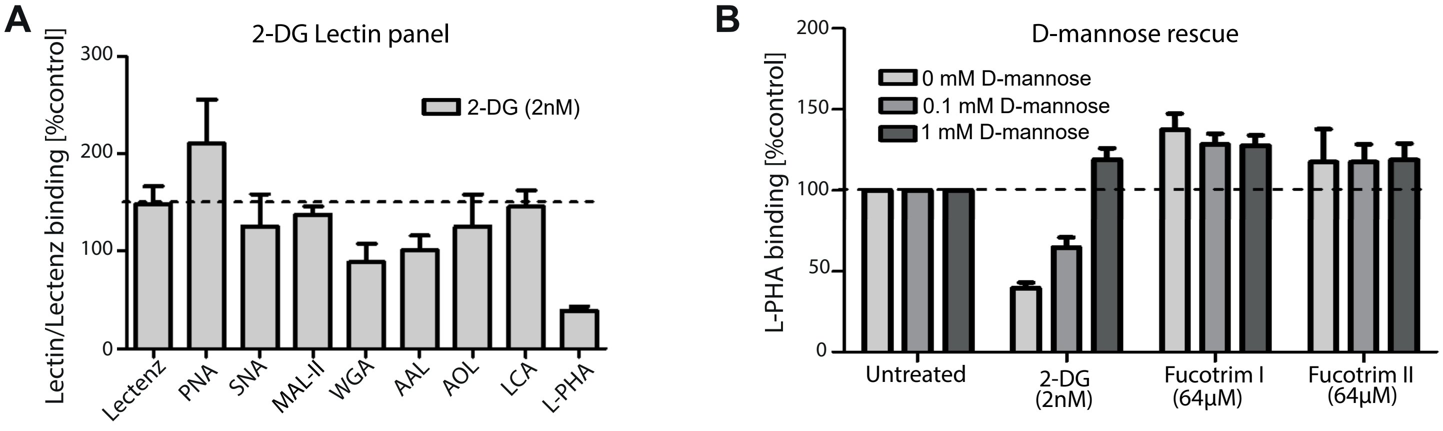

Supplementary Figure 11. PC3 cells mass spectrometry analysis of *N*-glycans

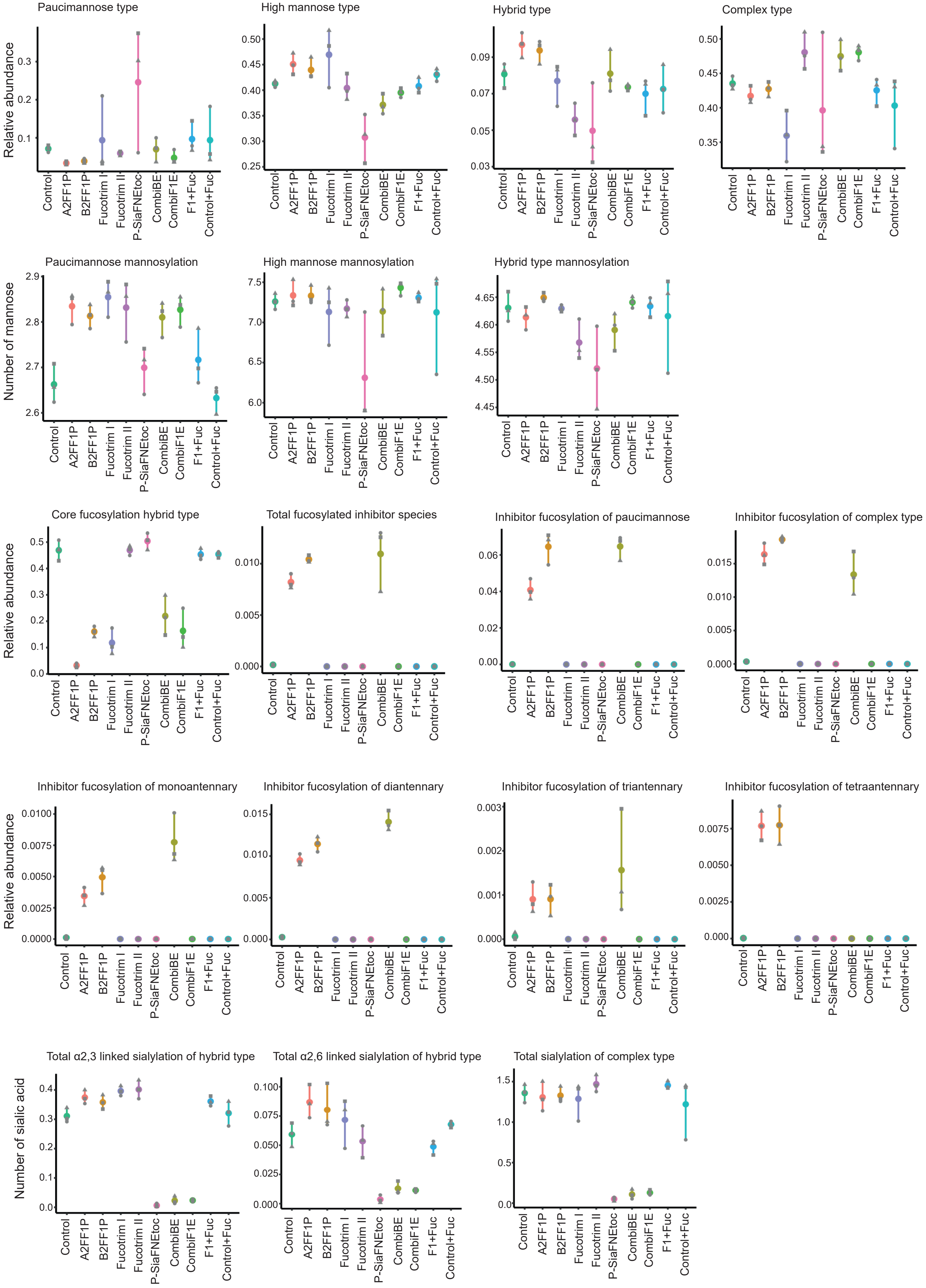

Supplementary Figure 12. PC3 cells mass spectrometry analysis of *O*-glycans

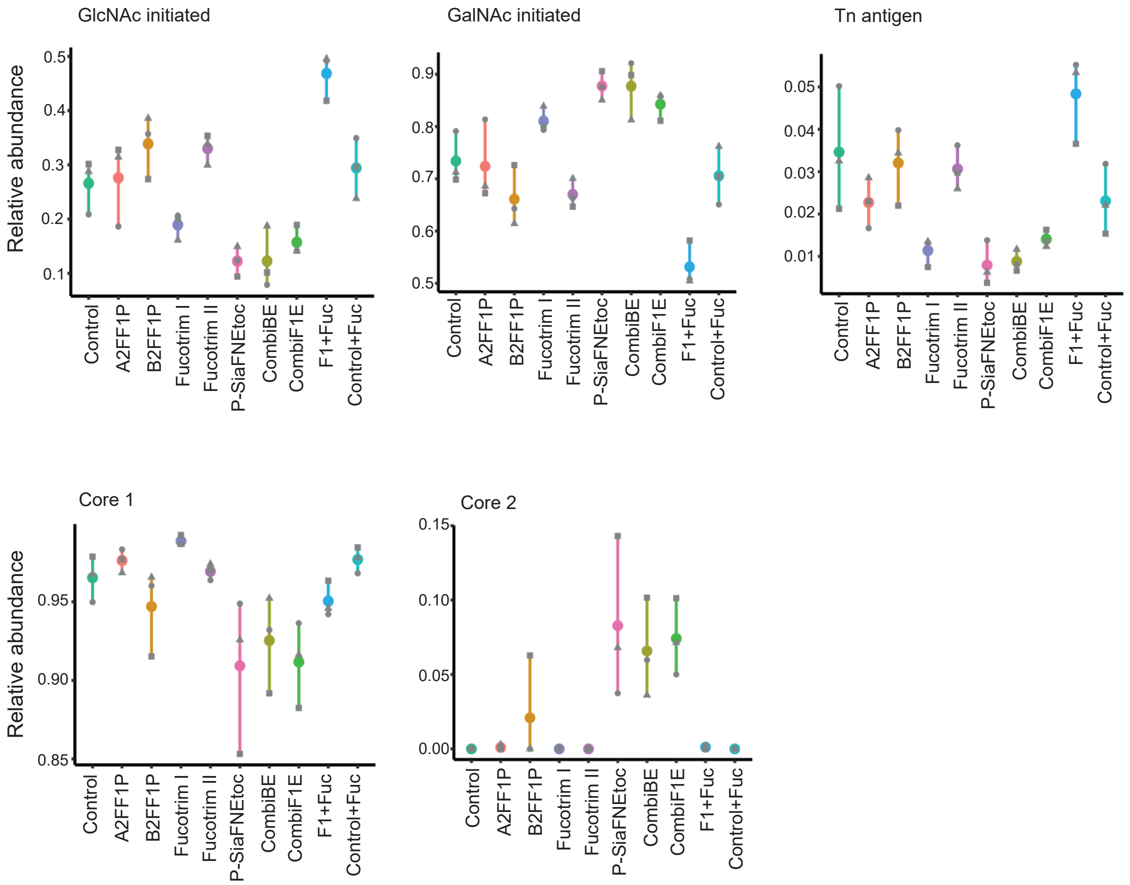

Supplement: Supplementary_Figures_cwad085 [file supplementary_figures_cwad085.zip › Supplementary_Figures_cwad085.pdf]
